# Chemical profiles and metabolite study of raw and processed *Cistanche deserticola* in rats by UPLC-Q-TOF-MS^E^

**DOI:** 10.1186/s13020-021-00508-0

**Published:** 2021-09-28

**Authors:** Zhe Li, Lkhaasuren Ryenchindorj, Bonan Liu, Ji Shi, Chao Zhang, Yue Hua, Pengpeng Liu, Guoshun Shan, Tianzhu Jia

**Affiliations:** 1 Pharmaceutic Department, Liaoning University of Traditional Chinese Medicine, Dalian, Liaoning China; 2 Drug Research Institute of Monos Group, Ulaanbaatar, 14250 Mongolia

**Keywords:** *Cistanche deserticola*, Processing, UPLC-Q-TOF-MS^E^, Chemical profiles, Metabolites in vivo

## Abstract

**Background:**

Chinese materia medica processing is a distinguished and unique pharmaceutical technique in Traditional Chinese Medicine (TCM) used for reducing side effects, and increasing or even changing therapeutic efficacy of the raw herbs.Changes in the essential components induced by an optimized processing procedure are primarily responsible for the increased efficacy of medicinal plants.The kidney-yang invigorating effect of rice wine-steamed *Cistancha deserticola* (*C. deserticola*) was stronger than raw *C. deserticola* (CD).

**Methods:**

A comparison analysis was carried out using the UPLC-Q-TOF-MS^E^ with the UNIFI informatics platform to determine the influence of processing. In vitro studies were performed for the characterization of constituents as well as metabolites in vivo. The chemical components were determined in CD and its processed products. The multivariate statistical analyses were conducted to evaluate variations between them while OPLS-DA was used for pairwise comparison.

**Results:**

The results of this study revealed considerable variations in phenylethanoid glycosides (PhGs) and iridoids after processing. A total of 97 compounds were detected in the extracts of CD and its processed product. PhGs having 4'-*O*-caffeoyl group in the 8-*O*-β-d-glucopyranosyl part, like acteoside, cistanoside C, campneoside II, osmanthuside decreased after being processed, while PhGs with 6'-*O*-caffeoyl group in the 8-*O*-β-d-glucopyranosyl part, such as isoacetoside, isocistanoside C, isocampneoside I, isomartynoside increased, especially in the CD-NP group. The intensity of echinacoside and cistanoside B whose structure possess 6'-*O*-β-d-glucopyranosyl moiety also increased. In in vivo study, 10 prototype components and 44 metabolites were detected in rat plasma, feces, and urine. The obtained results revealed that processing leads to the considerable variation in the chemical constituents of CD and affected the disposition of the compounds in vivo, and phase II metabolic processes are the key cascades of each compound and most of the metabolites are associated with echinacoside or acteoside.

**Conclusions:**

This is the first global comparison research of raw and processed CD. These findings add to our understanding of the impact of CD processing and give important data for future efficacy investigations.

## Introduction

*Chinese materia medica* (CMM) processing has demonstrated significant applicability in Traditional Chinese Medicine (TCM) clinical practice, and it has been considered a viable treatment for several centuries. This is a unique pharmaceutical technology that has been derived from the theory of TCM. Following processing, significant differences in the appearance, chemical constituents, characteristics, and medicinal significance of all types of TCMs have been identified, leading to the assumption that processing could improve the efficacy or reduce the TCM's toxic effects.

For hundreds of years, *Cistanche deserticola* (*Roucongrong* in Chinese, CD) is commonly used in TCM clinical practice for supplementing the functions of the kidney. It also helps in the moisturizing of the intestine that leads to relaxing bowel [1]. Cistanche was firstly recorded in *ShenNongBencaoJing*. It is commonly found in arid and semi-arid habitats across Eurasia and North Africa, including Iran, China, India, and Mongolia [2]. The processing of CD has been carried out by steaming with rice-wine under normal pressure, which is a preparation method documented in the Chinese pharmacopeia (*Jiucongrong* in Chinese, hereinafter called “CD-NP”). And CD steaming with rice-wine under high pressure is a more effective preparation method (hereinafter called “CD-HP”) [3, 4]. Several studies have been revealed that the pharmacological effects of CD are different from its processed products [5]. CD may tonify kidney-yang and relax bowel, while after being steamed by rice-wine, the effect of replenishing the kidney-yang would be strengthened. In our earlier study, it has been found that CD-NP could enhance tonification of the kidney and support *yang*, and relieve the effect of moistening intestines and defecating [6–8]. In clinical practice, the processed products are the most commonly used form.

Up to date, several studies have analyzed the chemical components of CD, followed by isolation and identification of more than 100 compounds [9–11], such as phenylethanol glycosides (PhGs), iridoids, lignans, and oligosaccharides as its main chemical constituents. It has also been reported that there are many pharmacological activities of PhGs including immunomodulatory, neuroprotective, hepatoprotective, anti-inflammatory, and anti-oxidative, etc.[12–14]. Iridoids possess anti-inflammatory activities [15, 16]. It has also been revealed by earlier studies that some chemical components showed variations during the processing [17–20]. Based on these reports, it can be assumed that post-processing, the variations in chemical composition lead to various pharmacological effects, which need to be further explored.

In the current study, a sensitive and effective method *i.e.,* ultra-high performance liquid chromatography coupled with TOF-MS^E^ (UPLC-Q-TOF-MS^E^) was performed for comparative analysis, and *in-vitro* studies were performed to qualitatively analyze the extracts of CD, CD-NP, and CD-HP for elucidating their chemical profiles. Generally, the exogenous chemicals with high exposure in target organs were regarded as effective components. Therefore, in rats, CD and its processed products were orally administered respectively, followed by their characterization. The existing study reveals a comparative study (both in vitro and in vivo) of raw and processed CD for the first time. The obtained results would expand our understanding regarding the effect of CD processing, which might be helpful for further studies.

## Materials and methods

### Materials

Standard compounds of ajugol (180120) and 2'-actylacetoside (M0601AS) were provided by Chendu Pure Chem-Standard Co., Ltd (Chengdu, China). Cistanoside F (MUST-17022620), echinacoside (D1105AS), cistanoside A (M0906AS), and isoacteoside (M0106AS) were provided by Must company (Sichuan China); acteoside (O0618AS), salidroside (J0526AS), catalpol (S0728AS), geniposide (A0407AS), and geniposidic acid (MB6001-S) were acquired from Dalian Meilun Bio.Co., Ltd (Dalian, China). 8-epideoxyloganic acid (B31123) was obtained from Shanghai Yuanye Biological Technology Co., Ltd, China. Methanol and acetonitrile were of MS-grade and were obtained from Merck KGaA, Darmstadt, Germany. Methanoic acid (CH_2_O_2_) of HPLC grade was provided by Merck KGaA (Darmstadt, Germany). The water, used in the existing study was processed via the Milli-Q system (18.2 MΩ, Millipore, Ma, USA). Rice-wine was provided by Brand Tower Shaoxing Wine Co., Ltd. (Zhejiang, China).

*Cistanch deserticola* was collected from *Neimenggu wangyedi* cistanche Co. Ltd. The samples were identified by Prof. Yanjun Zhai (school of pharmacy, *Liaoning University of TCM*). The specimens were submitted to the *Liaoning University of Traditional Chinese Medicine*.

### Animals

Sprague–Dawley male rats (SPF grade) with 180–220 g of total body weight were provided by Liaoning Changsheng biotechnology Co. Ltd. (Laboratory Animal Resource Center of Liaoning Province, license number: SCXK-2015–0001). These rats were housed in a breeding room with well-maintained temperature, and humidity *i.e.,* 20–26 ℃, 50–70% for one week. The rats were fed with usual lab food and water before experimentation. The animals fasted overnight, however, the water ad libitum was provided before the experimentation. The rats were executed with a 10% of chloral hydrate anesthetic. The Institutional Animal Ethics Committee of Liaoning Provincial Hospital of Chinese Medicine approved all of the experimental protocols (2019.3.25, 2019015).

### Preparation of CD, CD-NP, and CD-HP extract

CD-NP, CD-HP were processed from the same batch of *Cistanch deserticola*. To prepare CD-NP, dry CD pieces (5 mm thick, 100 g) were moisturized with rice-wine (30 mL) and were steamed at 100 ℃ for 16 h., followed by drying at 55 ℃ via drying oven. While CD-HP was prepared via infiltration of dry CD pieces (5 mm thick, 100 g) with rice-wine (30 mL), followed by steaming at 1.25 atmospheric pressure for 4 h. and then dried in a drying oven at 55 ℃.

In a 100 mL measuring flask, one gram of the powder was sieved via sieve#4, followed by adding 50% of methanol (50 mL) and then tightly covered and mixed. This mixture was weighed, followed by half hrs. maceration. After maceration, the mixture was ultrasonicated (power 250 W, frequency 35 kHz) for 40 min, followed by cooling, and weighing again. The loss of the weight was replenished with 50% methanol, properly mixed, and allowed to stand, followed by filtering the supernatant and then used the obtained filtrate as the test solution.

### MS^E^ analysis of active components

Preparation of standard substances: tubuloside-A (3.02 mg), echinacoside (3.00 mg), 2'-acetylacteoside (2.34 mg), acteoside (2.45 mg), isoacteoside (0.61 mg), cistanoside-F (2.14 mg), salidroside (3.39 mg), geniposide (2.84 mg), ajugol (1.58 mg), catalpol (2.39 mg), geniposidic acid (2.56 mg), and 8-epideoxyloganic acid (2.34 mg) were added into a 10 mL volumetric flask, added methanol constant volume to scale, configured into a corresponding concentration reference solution. Each of the 100 μL was configured into a mixed reference solution.

MS analysis condition: The mass value was corrected before the experiment, and the negative ion mode was used. The range of mass was 50–1200 Da, and the sample was injected through a flow injection pump. The cone velocity was 100 L/hrs, the dissolvent flow rate was set at 800 L/h. The capillary and cone voltages were fixed at 2500 and 40 V, accordingly. The temperature of the ion source and dissolvent gas was 100 ℃ and 400 ℃ respectively, and signal acquisition frequency was 0.5 S^−1^。

### UPLC-Q-TOF-MS^E^ analysis of CD extract

Chromatographic evaluations were carried out in a Waters ACQUITY I-CLASS UPLC system (Waters Corporation, Milford, MA, USA). Including ACQUITY UPLC® BEH C_18_ column (50 × 2.1 mm, 1.7 μm, Waters). The mobile phase was comprised of water having 0.1% formic acid (A) and acetonitrile contains 0.1% formic acid (B), the elution condition was as follows: 97% to 85% A (0–5 min), 85% to 75% A (5–15 min), 75% to 65% A (15–16 min), 65% to 55% A (16–18 min). The flow rate was 0.3 mL min^−1^, while the temperature of the auto-sampler room and column was 30 ℃ and 8 ℃ separately. The injection volume was 1.0 μL.

The mass spectrometric evaluation was carried out via Waters XEVO G2-XS QTOF MS (Waters Corporation, Milford, MA, USA), comprising an ESI source. The flow rate of nitrogen gas was fixed at 800 L·hrs^−1^ with a temp of 400 ℃, the source temp was fixed at 100 ℃, and the cone gas was set at 50 L h^−1^. The voltage of cone and capillary was adjusted at 40 and 2000 V, accordingly. The collision energy of the ramp was used in the range of 20–30 V. The centroided data of all samples were obtained from 50 to 1200 Da, with a 5-scan time of 0.5 s over an analysis time of 10 min. LockSpray TM was employed for the validation of the mass precision. The [M–H]^−^ ion of leucine enkephalin (200 pg·μL^−1^ infusion flow rate 10 μL min^−1^) at *m/z* 554.2615 was used as the lock mass. The MassLynx V4.1 software (Waters Co., Milford, USA) was employed for the accurate mass, the composition of the precursor ions, and the fragment ions calculation.

### Data analysis in Masslynx platform

Furthermore, an in-house library comprising the name of the compound, its structure, and the molecular formula (in mol.) was set up based on literature. All the compounds were noted in a special template, made in Excel. In addition, the mol files (Chemdraw Ultra 8.0, Cambridge soft, USA) and the Excel files of all the individual compound structures were also saved on the local PC. The established Excel-sheet having important data was directly imported into the scientific library in UNIFI.

UNIFI 1.8.2, Waters, Manchester, UK was employed for the evaluation of structural characteristics, particularly for the characteristic fragments and MS fragmentation. A minimum peak area of 500 was set for the 2D peak detection. During revealing 3D peaks, a low energy peak intensity of more than 300 counts and elevated energy peak intensity of more than 80 counts were chosen. The error of mass was found to be up to ± 10 ppm for known compounds, and the retention time tolerance was set in the range of ± 0.1 min. We selected the negative adducts containing –H, + HCOOH. The processing of the raw data obtained from MS was carried out via streamlined UNIFI software to rapidly pinpoint the chemical components that met the standards with the self-built database and the in-house Traditional Medicine Library.

Next, to verify the chemical structure of each target compound, the isomers were distinguished by their characteristic MS fragmentation patterns which were revealed in the reported studies, and by comparing the retention times of reference standards.

### Metabolomics analysis based on multivariate statistical analysis

Before processing the raw data, the parameters were set, such as mass ranging from 150 to 1200 Da, range of retention time (0 to 20 min), threshold intensity (2000 counts), mass tolerance *i.e.,* 5 mDa, while mass and retention time window was 0.20 min and 0.05 Da, respectively. In the subsequent list of the database, the identifier of ions was the RT-*m/z* pairs with respect to their elution times. The same values for RT and *m/z* in various batches of samples were considered as the same compound.

Multivariate statistical analysis was conducted to evaluate effective biomarkers that considerably contributed to variations among different groups. During the analysis, principal component analysis (PCA) was employed to indicate the maximum differences and pattern recognition for obtaining an overview and classification. The OPLS-DA is a modeling tool that provides visualization of the OPLS-DA predictive component loading to assist model evaluation. Variable importance for the projection (VIP) was used for assessing the evaluation of various components, and the metabolites with VIP values > 1.0 and *P-value* < 0.05 were regarded as effective markers. Furthermore, a permutation test was conducted for providing reference distributions for the R^2^/Q^2^ values that could show the statistical significance.

### Animal experiments

The rats were randomly categorized into four groups (n = 6 for each group), followed by the oral administration of various extracts: (1) Blank control group: the rats were given normal saline (2 mL/100 g); (2) CD group: the rats were given CD extract (2 mL/100 g); (3) CD-NP group: the rats were given CD-NP extract (2 mL/100 g); (4) CD-HP group: the rats were given CD-HP extract (2 mL/100 g). The further categorization of all groups was carried out into three sub-groups for plasma, urine, and feces, accordingly. Two hours later, each rat was orally administered with the same and equal amount of extracts.

Post administration, the collection of blood samples was carried out at 1.0 h, 2.0 h, and 4.0 h in heparinized 1.5 mL polythene tubes (from orbital veins), followed by centrifugation (at 4500 rpm) of all samples for 15 min.

For urine and feces samples, the rats were held in metabolism cages, and then the collection of urine and feces samples was carried out for 24 h after administration. The centrifugation of urine samples was carried out at 4500 rpm for 15 min, while feces samples were dried in the shade, ground into powder, then 0.2 g was taken, and added into 0.5 mL saline solution, ultrasound for 5 min, and centrifuged at 12,000 rpm for 15 min. All the bio-samples were kept at − 80 ℃ until analysis.

### Preparation of biological samples

The addition of plasma, urine, and feces samples was carried out with 3 volumes of methanol, followed by vortexing for 3 min. Next, the centrifugation (at 12,000 rpm) of the mixtures was carried out for 10 min, followed by transferring supernatant into the EP tube, and then dried by nitrogen at 37 ℃. Furthermore, the addition of 200 μL of HCN–H_2_O (50%) solution was carried out. Then, the vortex was used for mixing (1 min), followed by centrifugation (at 12,000 rpm) for 5 min. The supernatant (5 μL) of the treated samples was injected into the UPLC-Q-TOF-MS^E^ system.

### Liquid chromatographic and mass spectrometric condition

The analysis for metabolites was also performed by the Waters UPLC instrument through an ESI interface. Separations were carried out using an Acquity UPLC HSS T3 column (100 mm × 2.1 mm, 1.8 µm), the mobile phase was 0.1% formic acid (A): Acetonitrile (B), the gradient elution condition was 0–3 min (99.8% → 98% A), 3–5 min (98% → 95% A), 5–8 min (95% → 90% A), 8–12 min (90% → 85% A), 12–17 min (85% → 70% A), 17–22 min (70% → 60% A), 22–23 min (60% → 58% A), 23–25 min (58% A), 25–32 min (58% → 45% A), and 32–37 min (45% → 35% A), 0.4 mL min^−1^ was the flow rate. The temperature for the column and sample room was set at 40 ℃ and 8 ℃ respectively. The mass spectrometry conditions mentioned above were used.

### Strategy for systematic analysis of metabolites in bio-samples

UNIFI (1.8.2) software was employed for data processing. The Binary Compare function was used for the identification of effective metabolites. Evaluated metabolites were not existing in the equivalent control sample or exist at low ion intensities. The relative intensity threshold was set at 3 or 5, and metabolites that fulfilled the underlined criteria could be evaluated. Common and predictable metabolites were then determined by EIC. For searching of two-phase metabolites, the NLF function was applied. For example, in the UNIFI software, the parameters could be set at 176.0321 for searching for possible glucuronic acid conjugates. Post-processing, a neutral loss can be set in the method or identified. MassFragment was used for determining or characterization of detected metabolites' structures, a UNIFI’s spectral interpretation function is the main function used to analyze secondary fragmentation of parent components. This function can be used for rapid verification of the fragmentation path whether reasonable.

## Results

### Mass fragmentation rule of phenylethanoid glycosides and iridoids

**Fig. 1:**
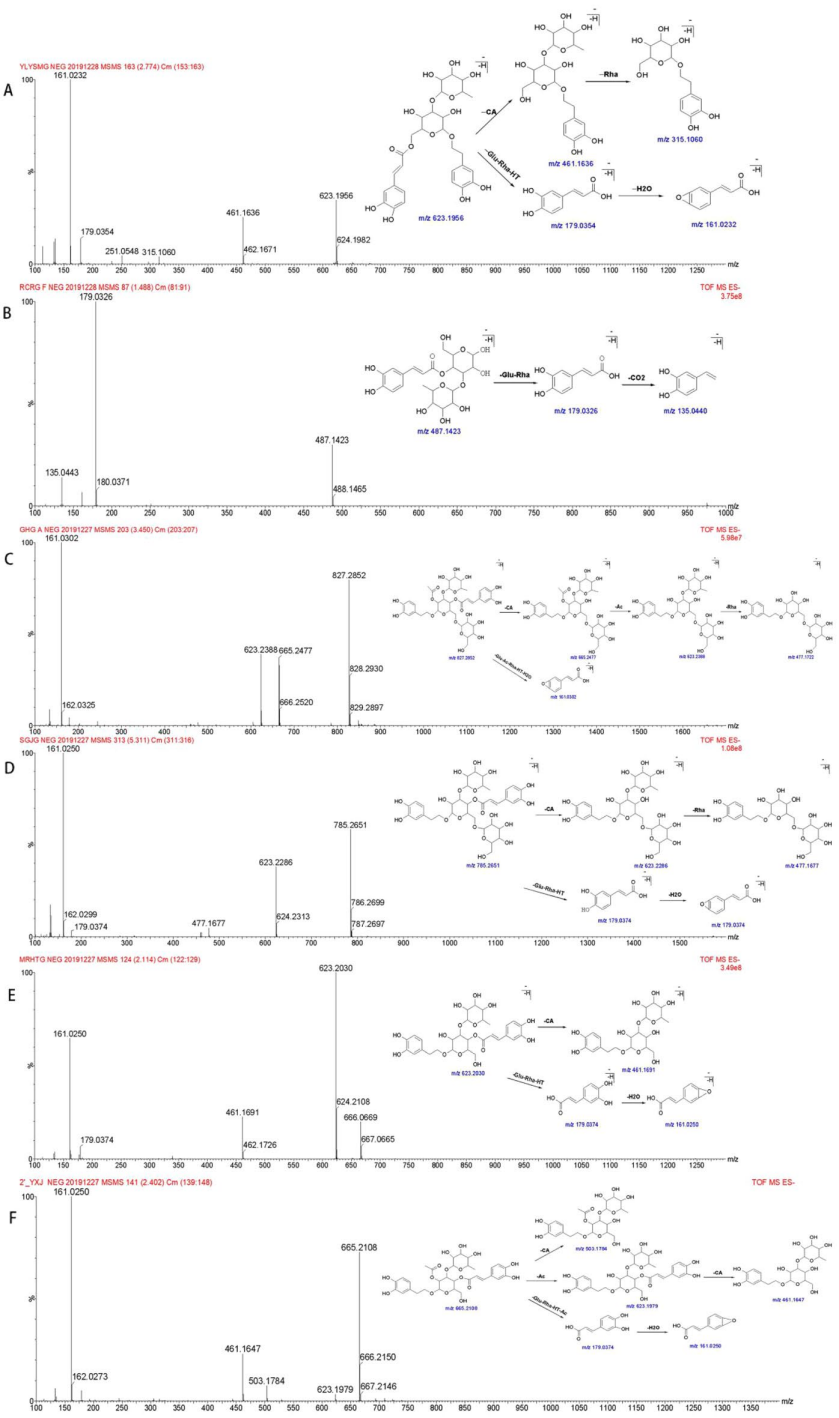
Mass Spectrogram and cleavage pathway of phenylethanoid glycosides. **A** Isoacteoside; **B **cistanosideF; **C** tubulosideA; **D** echinacteoside; **E** acteoside; **F **2'-actylacteoside

Phenylethanoid glycosides are the main chemical constituents of CD. The standard solutions of isoacteoside, cistanoside F, tubuloside A, echinacoside, acteoside, and 2'-actylacteoside were taken, followed by providing a different level of collision energies (Table 1), and then corresponding MS^2^ maps were obtained (Fig. 1).

**Table 1:** Collision energy for standard substances

Components	Molecular	Theoretical mass (Da)	Detected mass (Da)	Fragment	Energy (v)
Isoacteoside	C_29_H_36_O_15_	623.1976[M–H]^−^	623.1956[M–H]^−^	461.1636, 315.1636, 179.0354, 161.0232	35
Cistanoside F	C_21_H_28_O_13_	487.1452[M–H]^−^	487.1423[M–H]^−^	179.0326, 135.0440	20
Tubuloside A	C_37_H_48_O_21_	827.2610[M–H]^−^	827.2852[M–H]^−^	665.2477, 623.2388, 477.1722, 161.0202	40
Echinacoside	C_35_H_46_O_20_	785.2505[M–H]^−^	785.2651[M–H]^−^	623.2286, 477.1677, 179.0374, 161.0250	45
Acteoside	C_29_H_36_O_15_	623.1976[M–H]^−^	623.2030[M–H]^−^	461.1691, 179.0374, 161.0250	25
2'-actylacteoside	C_31_H_38_O_16_	665.2082[M–H]^−^	665.2108[M–H]^−^	623.1979, 503.1784, 461.1647, 179.0374, 161.0250	30
Ajugol	C_15_H_24_O_9_	347.1342[M–H]^−^	347.1410[M–H]^−^	185.0845, 167.0721, 149.0624, 127.0413	25
Catalpol	C_15_H_22_O_10_	361.1135[M–H]^−^	361.1131[M–H]^−^	199.0586, 169.0486, 151.0380, 125.0332	50
Geniposidic acid	C_16_H_22_O_10_	373.1135[M–H]^−^	373.1143[M–H]^−^	211.0602, 193.0500, 149.0608, 167.0703, 123.0453	30
Geniposide	C_17_H_24_O_10_	387.1291[M–H]^−^	387.1313[M–H]^−^	225.0787, 207.0678, 123.0453	10
8-epideoxyloganic acid	C_16_H_24_O_9_	359.1342[M–H]^−^	359.1345[M–H]^−^	197.0810, 153.0916, 135.0823	40

The mass spectrometric analysis revealed that phenylethanoid glycosides have similar mass spectrum fragmentation patterns, the cleavage pathways in the negative-ion mode mainly include: (1) Ester bond cleavage: loss of neutral caffeoyl group (C_9_H_3_O_6_, 162.03) and neutral acetyl group (C_2_H_2_O, 42.00); (2) Glycosidic cleavage: loss of neutral rhamnose residues (C_6_H_10_O_4_, 146.05) and neutral glucose residue (C_6_H_10_O_5_, 162.05). From high-resolution mass spectrometry, caffeoyl (162.03) and glucose residue (162.05) could be distinguished.

**Fig. 2:**
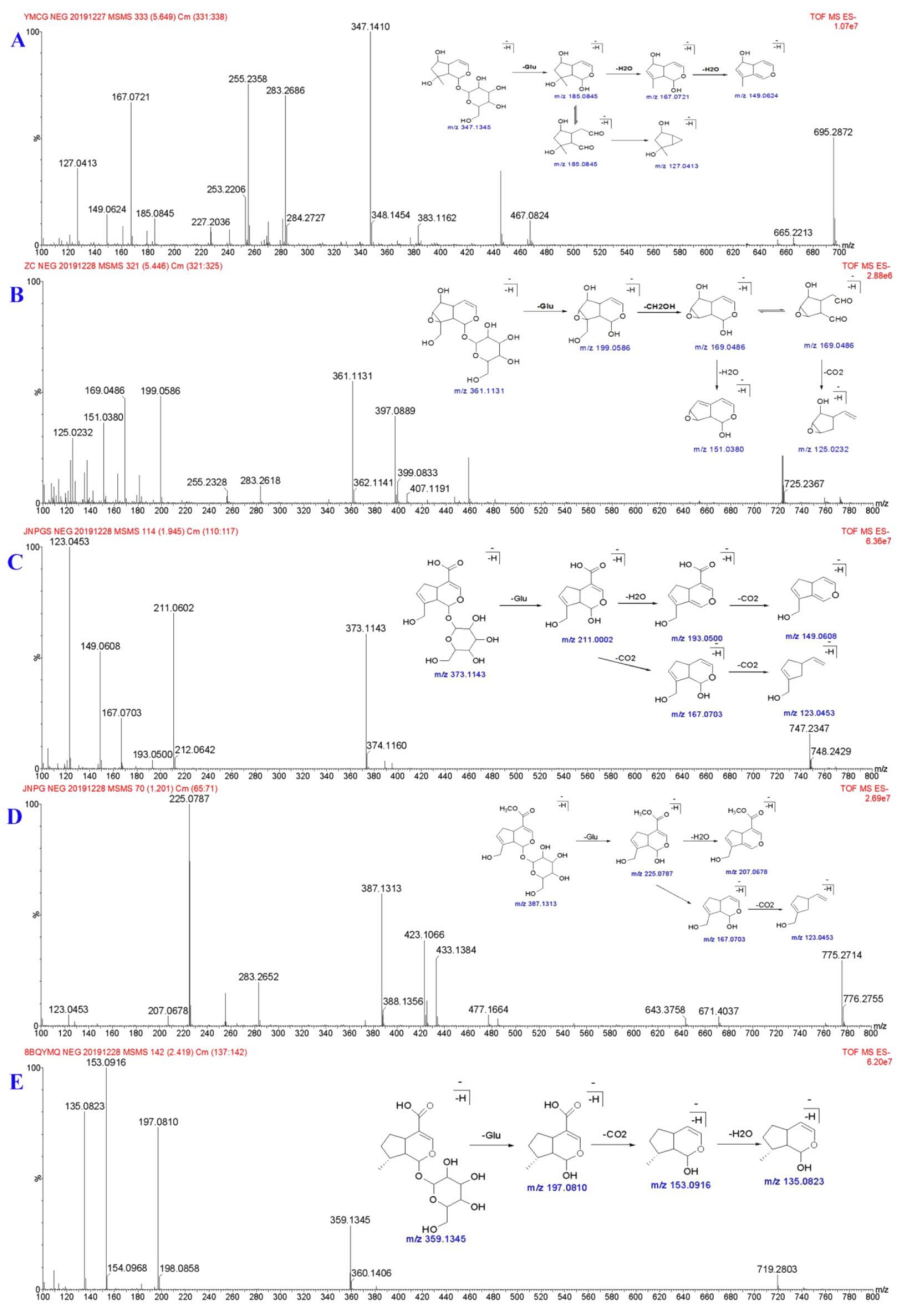
Mass Spectrogram and cleavage pathway of iridoid glycosides. **A** Ajugol, **B** catalpol, **C** geniposidic acid, **D** geniposide, **E **8-epideoxyloganic acid

Iridoids ajugol, catalpol, geniposidic acid, geniposide, and 8-epideoxyloganic acid standard solutions were taken, followed by providing different collision energies, and corresponding MS^2^ maps were obtained (Fig. 2).

Iridoid glycosides have similar mass spectrum fragmentation patterns, the cleavage pathways in the negative-ion mode mainly include (1) Glycosidic cleavage: Loss of neutral glucose residue (C_6_H_10_O_5_, 162.05); (2) Loss of neutral CO_2_ (43.99) and H_2_O (18.01).

### Identification of the compounds in CD, CD-NP, and CD-HP extracts

#### ***UPLC-QTOF-MS***^***E***^*** analysis***

**Fig. 3:**
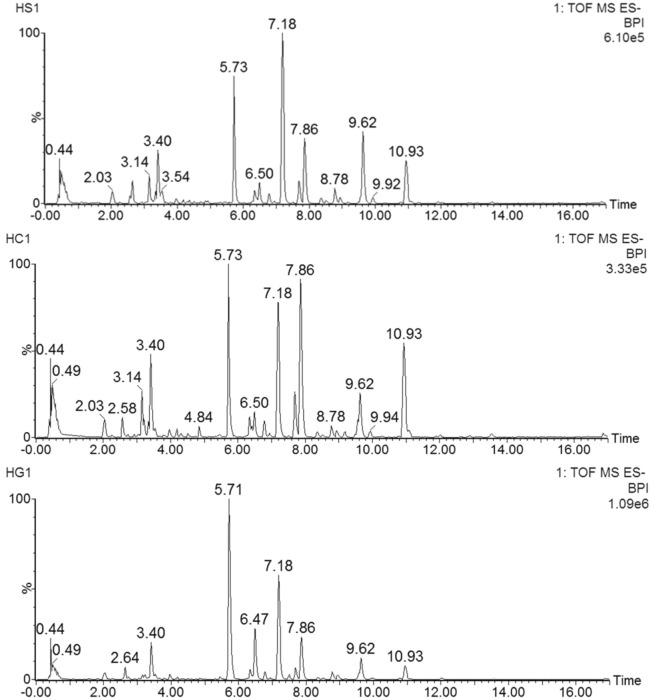
The base peak intensity (BPI) of the samples. 1.CD, 2. CD-NP, 3. CD-HP

The optimization of chromatographic conditions was carried out. Next, the compounds of Cistanche Herba were evaluated in both negative and positive ion modes with high as well as low CEs. The obtained results revealed that the compatibility of the negative mode was higher relative to the positive mode for these compounds. Figure 3 showed MS basic peak ion (BPI) chromatogram traced with numbered peaks. The intensity of each detected ion in UPLC-Q-TOF-MS^E^ analysis was normalized with respect to the whole ion count for the generation of a data matrix which comprised of *m/z* value, the normalized peak area, and retention time.

### The evaluation of components from CD and its processed products on the UNIFI platform

A total of 97 compounds were identified with -SEM (n = 6) mode from CD and its processed product (Table 2), including phenylethanoid glycosides (PhGs), iridoids, lignans, and oligosaccharides. The 95, 91, and 94 components were detected in CD, CD-NP, and CD-HP, accordingly. Among them, 64 were phenylethanoids, 13 were iridoids, and 20 other kinds of compounds were determined. There was a similarity in the chemical composition of CD and its processed product, however, the quantity of the components was found to be different among CD and its processed product.

**Table 2:** Evaluation of Compounds obtained from CD and its processed products by UPLC-Q-TOF-MS^E^

NO	RT	Identification	Molecular formula	Adducts	Experimental	Theoretical	Error (ppm)	MS/MS fragmentation	source
1	1.10	Kankanoside B	C_15_H_24_O_10_	+ HCOO	409.1348	364.1369	0.2	363.12870, 183.06693, 153.05619	CD, CD-HP
2	1.22	6-Deoxycatalpol	C_15_H_22_O_9_	+ HCOO	391.1245	346.1260	0.5	391.12447, 341.10886,	CD, CD-NP, CD-HP
3	1.44	6-Deoxycatalpol	C_15_H_22_O_9_	+ HCOO	391.1245	346.1260	0.5	391.12448, 183.06662	CD, CD-HP
4	2.04	Androsin	C_15_H_20_O_8_	+ HCOO	373.1143	328.1163	0.8	373.11434, 211.06188, 193.05142	CD, CD-NP, CD-HP
5	2.25	6-Deoxycatalpol	C_15_H_22_O_9_	+ HCOO	391.1245	346.1260	0.5	391.12446, 229.07232, 167.03571	CD, CD-NP, CD-HP
6	2.58	Androsin	C_15_H_20_O_8_	+ HCOO	373.1147	328.1166	1.2	373.11471, 299.11294, 211.06206, 149.06137	CD, CD-NP, CD-HP
7	2.64	Kankanoside L	C_15_H_24_O_9_	+ HCOO	393.1404	348.1421	0.7	393.14046, 315.10749, 206.06863, 134.04736	CD, CD-NP, CD-HP
8	2.93	kankanoside M	C_15_H_22_O_8_	+ HCOO	375.1297	330.1318	0.6	375.12966, 213.07775, 125.06127	CD, CD-NP, CD-HP
9	3.14	3,4-dimethoxybenzyl-β-d-glucoside	C_16_H_24_O_10_	–H	375.1299	376.1373	0.8	375.12994, 255.08683, 213.07767, 151.07707	CD, CD-NP, CD-HP
10	3.22	Decaffeoylacteoside	C_20_H_30_O_12_	–H	461.1659	462.1734	0	461.16591, 315.10891, 135.04591	CD, CD-NP, CD-HP
11	3.24	Kankanoside F	C_26_H_40_O_17_	–H	623.2192	624.2273	0.5	623.21920, 461.16678, 315.10994, 135.04591	CD, CD-NP, CD-HP
12	3.25	Gluroside	C_15_H_24_O_8_	+ HCOO	377.1449	332.1463	0.1	377.14491, 461.16609, 315.10891, 135.04591	CD, CD-NP, CD-HP
13	3.31	Cistantubulose A1	C_27_H_38_O_18_	–H	649.1987	650.2068	0.7	649.19871, 537.18251, 335.09146, 179.03598,	CD, CD-NP, CD-HP
14	3.35	6-deoxycatalpol	C_15_H_22_O_9_	–H	345.1193	346.1271	0.7	345.11929, 299.11424, 179.03598	CD, CD-NP, CD-HP
15	3.40	Adoxosidic acid	C_16_H_24_O_10_	–H	375.1302	376.1373	− 1.1	213.07683	CD, CD-NP, CD-HP
16	3.54	Cistanoside F	C_21_H_28_O_13_	–H	487.1451	488.1532	− 0.1	487.14512, 325.09503, 251.05822, 179.03637	CD, CD-NP, CD-HP
17	3.65	Sinapic aldehyde 4-O-β-d-glucopyranoside	C_16_H_20_O_9_	+ HCOO	401.1087	356.1109	0.3	401.10872, 301.09,397, 283.08371, 193.05148	CD, CD-NP, CD-HP
18	3.77	Kankanose	C_27_H_38_O_18_	–H	649.1985	650.2063	0.5	649.19853, 461.16139, 293.12495, 179.03612	CD, CD-NP, CD-HP
19	3.79	3-Methyl-but-2-en-1-yl-β-d-glucopyranoside	C_11_H_20_O_6_	+ HCOO	293.1249	248.1264	1.3	293.12494, 195.06673, 179.03612	CD, CD-NP, CD-HP
20	3.81	Demethylsyringing	C_16_H_22_O_9_	–H	357.1193	358.1266	0.7	357.11931, 251.05778, 195.06,678, 179.03653	CD, CD-NP, CD-HP
21	3.82	Cistanoside G	C_20_H_30_O_11_	–H	445.1709	446.1756	− 0.1	445.17092, 375.13013, 293.12548, 195.06823, 179.03678	CD, CD-NP, CD-HP
22	3.84	Cistanoside F	C_21_H_28_O_13_	–H	487.1458	488.1523	− 0.6	487.14577, 445.17102,323.08273, 179.03678,	CD, CD-NP, CD-HP
23	3.96	3-Methyl-but-2-en-1-yl-β-D-glucopyranoside	C_11_H_20_O_6_	+ HCOO	293.1251	248.1268	1.5	293.12505, 161.04759	CD, CD-NP, CD-HP
24	3.98	Gluroside	C_15_H_24_O_8_	+ HCOO	377.1455	332.1476	0.7	377.14547, 293.12505, 179.03614	CD, CD-HP
25	4.03	Cistanoside F	C_21_H_28_O_13_	–H	487.1457	488.1538	0.5	487.14668, 2,23.06196, 179.03779	CD, CD-NP, CD-HP
26	4.16	Kankanoside D	C_15_H_26_O_7_	+ HCOO	363.1663	318.1679	0.8	363.16634, 315.10883, 179.03855, 161.04465	CD, CD-NP, CD-HP
27	4.19	Cistanoside E	C_21_H_32_O_12_	–H	475.1869	476.1888	5.3	475.18694, 363.16559, 179.03,855	CD, CD-NP, CD-HP
28	4.25	Cistanoside I	C_21_H_28_O_12_	–H	471.1505	472.1577	0.2	471.15048, 369.11987, 471.15071, 179.03589, 163.04110	CD, CD-NP, CD-HP
29	4.32	Cistanoside F	C_21_H_28_O_13_	–H	487.1466	488.153	1.4	487.14656, 323.07922, 251.05793, 179.03699	CD, CD-NP, CD-HP
30	4.53	Cistanoside F	C_21_H_28_O_13_	–H	487.1464	488.1537	1.2	487.14637, 323.08028, 251.05805, 179.03769	CD, CD-NP, CD-HP
31	4.55	Androsin	C_15_H_20_O_8_	–H	327.1092	328.1158	1.2	327.10918, 251.05805, 179.03769, 131.07201	CD, CD-NP, CD-HP
32	4.86	Cistanoside H	C_22_H_32_O_13_	–H	503.1761	504.1835	− 0.4	503.17610, 461.16590, 375.13036, 315.10972, 135.04603	CD, CD-NP, CD-HP
33	4.88	Kankanoside E	C_16_H_28_O_8_	+ HCOO	393.1760	348.1784	− 0.1	393.17603, 241.11923, 375.13036, 161.03814	CD, CD-HP
34	4.92	Cistantubuloside C 1 /C 2	C_35_H_46_O_21_	–H	801.2487	802.2556	3.4	801.24867, 623.20258, 110.03824	CD, CD-NP, CD-HP
35	5.29	(2E,6Z)-2-β-d-Glucopyranosyloxy-2,6-dimethyl-2,6-octadienoic acid	C_16_H_26_O_8_	+ HCOO	391.1608	346.1628	0.4	391.16080, 345.15509, 163.03730	CD, CD-NP, CD-HP
36	5.47	Kankanoside E	C_16_H_28_O_8_	+ HCOO	393.1766	348.1783	0.5	393.17656, 283.07834, 179.03768	CD, CD-NP, CD-HP
37	5.56	Campneoside II	C_29_H_36_O_16_	–H	639.1926	640.2003	0.1	621.19264, 361.15007, 161.02704	CD, CD-NP, CD-HP
38	5.73	Echinacoside	C_35_H_46_O_20_	–H	785.2549	786.2618	4.5	785.25485, 623.21903, 392.11650, 179.03596	CD, CD-NP, CD-HP
39	5.81	8-hydroxygeraniol-1-β-d-glucopyranoside	C_16_H_28_O_7_	+ HCOO	377.1813	332.1835	0.1	377.18129, 331.14023, 164.07382	CD, CD-NP, CD-HP
40	5.86	Cistanoside E	C_21_H_32_O_12_	–H	475.1813	476.1888	− 0.3	347.17188, 251.05915	CD, CD-NP, CD-HP
41	5.93	Liriodendrin	C_34_H_46_O_18_	+ HCOO	787.2670	742.2689	0.9	787.26703, 579.20978, 475.18047, 417.15414	CD, CD-NP
42	6.00	Isolariciresinol-9′-O-β-d-glucopyranoside	C_26_H_34_O_11_	+ HCOO	567.2076	522.2095	− 0.2	567.20755, 359.14970, 329.13966, 178.06231	CD, CD-NP, CD-HP
43	6.06	Campneoside II	C_29_H_36_O_16_	–H	639.1936	640.2003	1.1	639.19362, 487.14472, 251.05630	CD, CD-NP, CD-HP
44	6.26	Kankanosides K 1 /K 2	C_36_H_48_O_21_	–H	815.2636	816.2701	2.6	815.26358, 783.23518, 637.1979, 381.15558, 179.03631	CD, CD-NP, CD-HP
45	6.34	Cistantubuloside B 1	C_35_H_46_O_19_	–H	769.2573	770.2655	1.8	769.25732, 623.21303, 420.06489, 163.03926	CD, CD-NP, CD-HP
46	6.36	8-hydroxygeraniol-1-β-d-glucopyranoside	C_16_H_28_O_7_	+ HCOO	377.1820	332.1833	0.8	377.18204, 367.15243, 163.04196	CD, CD-NP, CD-HP
47	6.42	Kankanoside N	C_16_H_26_O_8_	–H	345.1563	346.1637	1.4	345.15630, 197.80891, 113.02490	CD, CD-NP, CD-HP
48	6.50	Cistanoside A	C_36_H_48_O_20_	+ HCOO	845.2769	800.2781	5.4	845.27689, 799.27001,681.20502	CD, CD-NP, CD-HP
49	6.47	Kankanoside I	C_35_H_46_O_18_	+ HCOO	799.2703	754.2718	4.2	799.27031, 365.08428, 161.02522	CD, CD-NP, CD-HP
50	6.79	(2E,6E)-2-β-d-glucopyranosyloxy-2,6-dimethyl-2,6-octadienoic acid	C_16_H_26_O_8_	–H	345.1565	346.1632	1.6	345.15649, 165.09327	CD, CD-NP, CD-HP
51	6.96	Kankanoside A	C_16_H_26_O_8_	–H	345.1565	346.1633	1.6	345.15647, 195.06666, 179.03628	CD, CD-NP, CD-HP
52	7.03	Cistanoside C	C_30_H_38_O_15_	+ HCOO	683.2198	638.2208	1.1	683.21978, 489.14915, 417.15349, 335.20636, 197.80796	CD, CD-NP, CD-HP
53	7.09	kankanoside E	C_16_H_28_O_8_	–H	347.1716	348.1781	1.0	347.17157, 195.81307, 167.10929	CD, CD-NP, CD-HP
54	7.19	Acteoside	C_29_H_34_O_15_	–H	623.1992	622.1892	1.6	623.19917, 461.16657, 315.10988, 161.02530	CD, CD-NP, CD-HP
55	7.25	Tubuloside A	C_37_H_48_O_21_	–H	827.2655	828.2721	4.5	827.26548, 621.18343, 469.13652, 379.19635	CD, CD-NP, CD-HP
56	7.51	Cistanoside B	C_37_H_50_O_20_	+ HCOO	859.2913	814.2931	4.1	859.2913, 679.18910, 565.19246	CD, CD-NP, CD-HP
57	7.58	Cistanoside J	C_33_H_42_O_16_	+ HCOO	739.2409	694.2482	4.0	739.24093, 345.15468, 161.02597	CD, CD-NP, CD-HP
58	7.60	Tubuloside A	C_37_H_48_O_21_	–H	827.2649	828.2727	3.9	827.26486, 739.24745, 579.22756, 345.15468, 161.02597	CD-NP, CD-HP
59	7.7	Kankanoside E	C_16_H_28_O_8_	–H	347.1719	348.1791	1.3	347.17191, 303.18323, 211.13616, 185.11917,	CD, CD-NP, CD-HP
60	7.86	Acteoside	C_29_H_36_O_15_	–H	623.1995	624.2067	1.9	623.19954, 461.16624, 161.02546	CD, CD-NP, CD-HP
61	7.94	Crenatoside	C_29_H_34_O_15_	–H	621.1833	622.1907	1.4	621.18331, 387.14418, 179.03640	CD, CD-NP, CD-HP
62	8.06	Kankanosides K1/K2	C_36_H_48_O_21_	–H	815.2631	816.2688	2.1	499.1811, 197.8080, 160.8423	CD-HP
63	8.33	Kankanoside H1	C_37_H_48_O_20_	–H	812.2731	812.2739	− 0.8	607.20431, 445.17033, 161.02556	CD, CD-HP
64	8.36	Isosyringalide-3´-α-L-rhamnopyranoside	C_29_H_36_O_14_	–H	607.2034	608.2119	0.7	607.20341, 461.16447, 315.10906, 145.03063	CD, CD-NP, CD-HP
65	8.53	Campneoside I	C_30_H_38_O_16_	–H	653.2084	654.216	1.6	607.20440, 461.16367, 443.15204, 145.03081	CD, CD-NP, CD-HP
66	8.78	Cis-isocistanoside C	C_30_H_38_O_15_	–H	637.2147	638.2222	1.5	637.21474, 475.18074, 329.12012, 161.02576	CD, CD-NP, CD-HP
67	8.84	Citrusin A	C_27_H_36_O_11_	+ HCOO	581.2235	536.2280	0.1	581.22351, 433.15241,371.13360,343.1457	CD, CD-NP, CD-HP
68	9.17	Isosyringalise-3´-ɑ-L-rhamnopyranoside	C_29_H_36_O_14_	–H	607.2033	608.2122	0.6	607.20334, 461.15822, 161.02611	CD, CD-NP, CD-HP
69	9.50	Isocampneoside I	C_30_H_38_O_16_	–H	653.2094	654.2161	1.2	607.2094, 461.16616, 307.08417, 145.03058	CD, CD-NP, CD-HP
70	9.50	Syringalide A-3´-α-L-rhamnopyranoside	C_29_H_36_O_14_	–H	607.2037	608.2127	1.0	607.20372, 461.16616, 307.08417, 145.03089	CD, CD-NP, CD-HP
71	9.57	isocistanoside C	C_30_H_38_O_15_	–H	637.2150	638.2221	1.8	637.21503, 445.15153, 323.07862, 251.05653	CD, CD-NP, CD-HP
72	9.62	Cis-Tubuloside B	C_31_H_38_O_16_	–H	665.2103	666.2169	2.1	665.21032, 503.17680, 305.06585, 161.02529	CD, CD-NP, CD-HP
73	9.76	Crenatoside	C_29_H_34_O_15_	–H	621.1826	622.1891	0.7	621.18264, 487.14611, 323.07878, 179.03579	CD, CD-NP, CD-HP
74	9.92	cistanoside C	C_30_H_38_O_15_	–H	637.2139	638.2209	0.7	637.21389, 591.20868, 445.16991, 163.04078, 145.03032	CD, CD-NP, CD-HP
75	9.92	Osmanthuside B	C_29_H_36_O_13_	–H	591.2080	592.2158	0.2	591.20804, 445.16991, 160.84291, 145.03032	CD, CD-NP, CD-HP
76	10.28	Eutigoside A	C_23_H_26_O_9_	–H	445.1501	446.1571	0.2	445.15008, 163.03943, 145.03004	CD, CD-NP, CD-HP
77	10.35	Cistanoside M	C_30_H_38_O_14_	+ HCOO	667.2245	622.2248	0.7	667.22446, 621.21761, 555.20753, 161.02534	CD, CD-NP, CD-HP
78	10.78	Isomartynoside	C_31_H_40_O_15_	+ HCOO	697.2356	652.2372	1.2	697.23563, 651.22859, 475.17960, 175.04062	CD, CD-NP, CD-HP
79	10.92	Salsaside B	C_28_H_34_O_13_	–H	577.1929	578.1999	0.8	503.17758, 323.07755, 161.02527	CD, CD-NP, CD-HP
80	10.93	2´-acetylacteoside	C_31_H_38_O_16_	–H	665.2108	666.2173	2.6	665.21076, 503.17758, 305.06712, 161.02527	CD, CD-NP, CD-HP
81	11.08	Osmanthuside B	C_29_H_36_O_13_	–H	591.2084	591.2093	0.6	445.1579, 163.0400, 145.0301	CD, CD-NP, CD-HP
82	11.08	Plantainoside C	C_30_H_38_O_15_	–H	637.2141	638.2238	0.9	591.20986, 445.15921, 145.03022	CD, CD-NP, CD-HP
83	11.33	Kankanosides J1/J2	C_32_H_40_O_17_	–H	695.2190	696.2267	0.3	695.21902, 649.21477, 503.17505, 145.03017	CD, CD-NP, CD-HP
84	11.33	SalsasideF	C_31_H_38_O_15_	–H	649.2140	650.2199	0.8	649.21399, 503.17505, 347.16994, 145.03017	CD, CD-NP, CD-HP
85	11.89	Cistansinenside A	C_32_H_40_O_16_	–H	679.2246	680.2324	0.8	679.22464, 623.19749, 161.02503	CD, CD-NP, CD-HP
86	12.02	isomartynoside	C_31_H_40_O_15_	+ HCOO	697.2360	652.2364	1.6	697.23604, 651.22862, 505.16921, 175.04095	CD, CD-NP, CD-HP
87	12.63	Salsaside A	C_28_H_34_O_13_	–H	577.1932	578.1982	1.1	577.19316, 501.16521, 469.13425, 179.03540, 161.02496	CD, CD-NP, CD-HP
88	12.84	Salsaside Ca/Cb	C_28_H_34_O_12_	–H	561.1978	562.2044	0.6	561.19776, 415.16021, 163.04118, 145.03011	CD, CD-NP, CD-HP
89	12.90	SalsasideF	C_31_H_38_O_15_	–H	649.2144	650.2212	1.2	649.21443, 503.17434, 461.16538	CD, CD-NP, CD-HP
90	12.90	Kankanosides J1/J2	C_32_H_40_O_17_	–H	695.2195	696.2274	0.8	695.21948, 649.21421, 607.20459, 503.17401, 149.02394	CD, CD-NP, CD-HP
91	13.10	Osmanthuside B	C_29_H_36_O_13_	–H	591.2085	592.2162	0.7	591.20845, 429.17830, 161.02556	CD, CD-NP, CD-HP
92	13.11	Jionoside D	C_30_H_38_O_15_	–H	637.2131	638.2210	− 0.1	591.20842, 161.02556	CD, CD-NP, CD-HP
93	13.17	Salsaside D	C_31_H_38_O_15_	–H	649.2140	650.2199	0.8	649.21401, 607.19810, 329.16152	CD, CD-NP, CD-HP
94	13.53	cistansinenside A	C_32_H_40_O_16_	–H	679.2251	680.2314	1.3	679.22512, 637.21464, 461.16791, 161.02601	CD, CD-NP, CD-HP
95	14.36	Osmanthuside B6(Z)	C_29_H_36_O_13_	–H	591.2085	592.2152	0.7	591.20854, 489.26955, 445.15395, 161.02652	CD, CD-NP, CD-HP
96	15.30	sinenside A	C_32_H_40_O_16_	–H	679.2255	680.2328	1.7	679.22547, 633.22252, 591.20916, 145.03024	CD, CD-NP
97	16.43	Cistanoside M	C_30_H_38_O_14_	–H	621.2184	622.2258	0.1	591.20868, 489.27111, 161.02535	CD, CD-NP

### Variations in chemical components of processed products

**Fig. 4:**
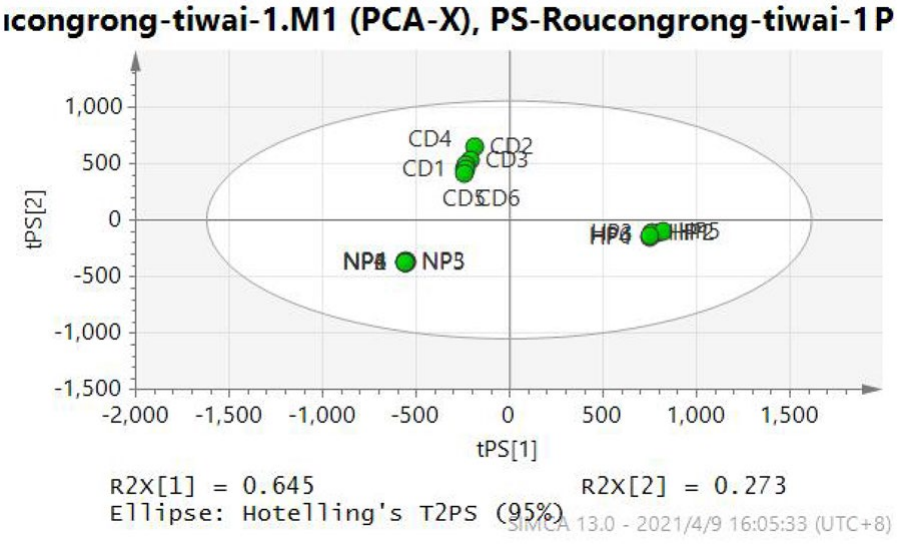
The PCA of CD and its different processed products

The Simca-P 13.0 software was employed for analyzing the multivariate data matrix. Before PCA, all variables were mean-centered and pareto-scaled, followed by identification of potential discriminant variables. In a PCA score plot, every point showed an individual sample. Samples that showed similarity in their chemical components were scattered adjacent to each other, while those which showed variations in their components were divided. As seen in PCA (Fig. 4), the group of CD-HP was separated from the groups of CD and CD-NP.

**Fig. 5:**
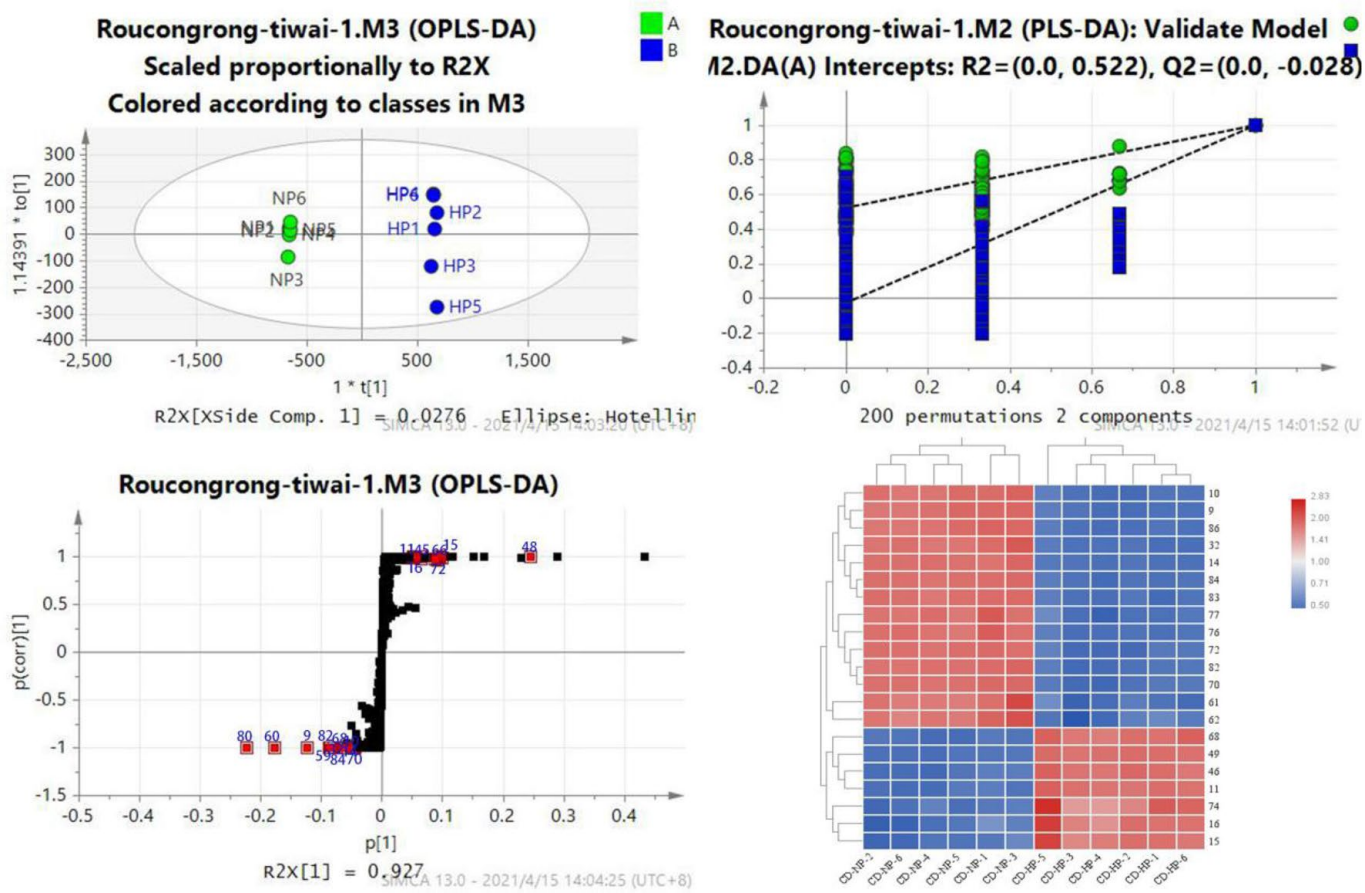
The OPLS-DA/permutation test/S-plot/heat map indicating the intensities of potential biomarkers between CD-NP and CD-HP Compounds 9, 10, 14, 32, 59, 60, 68, 70, 74, 75, 80, 81, 82, and 84 are the differential components of CD-NP, while compounds 11, 15, 16, 45, 48, 66, and 72 are the differential components of CD-HP.

**Fig. 6:**
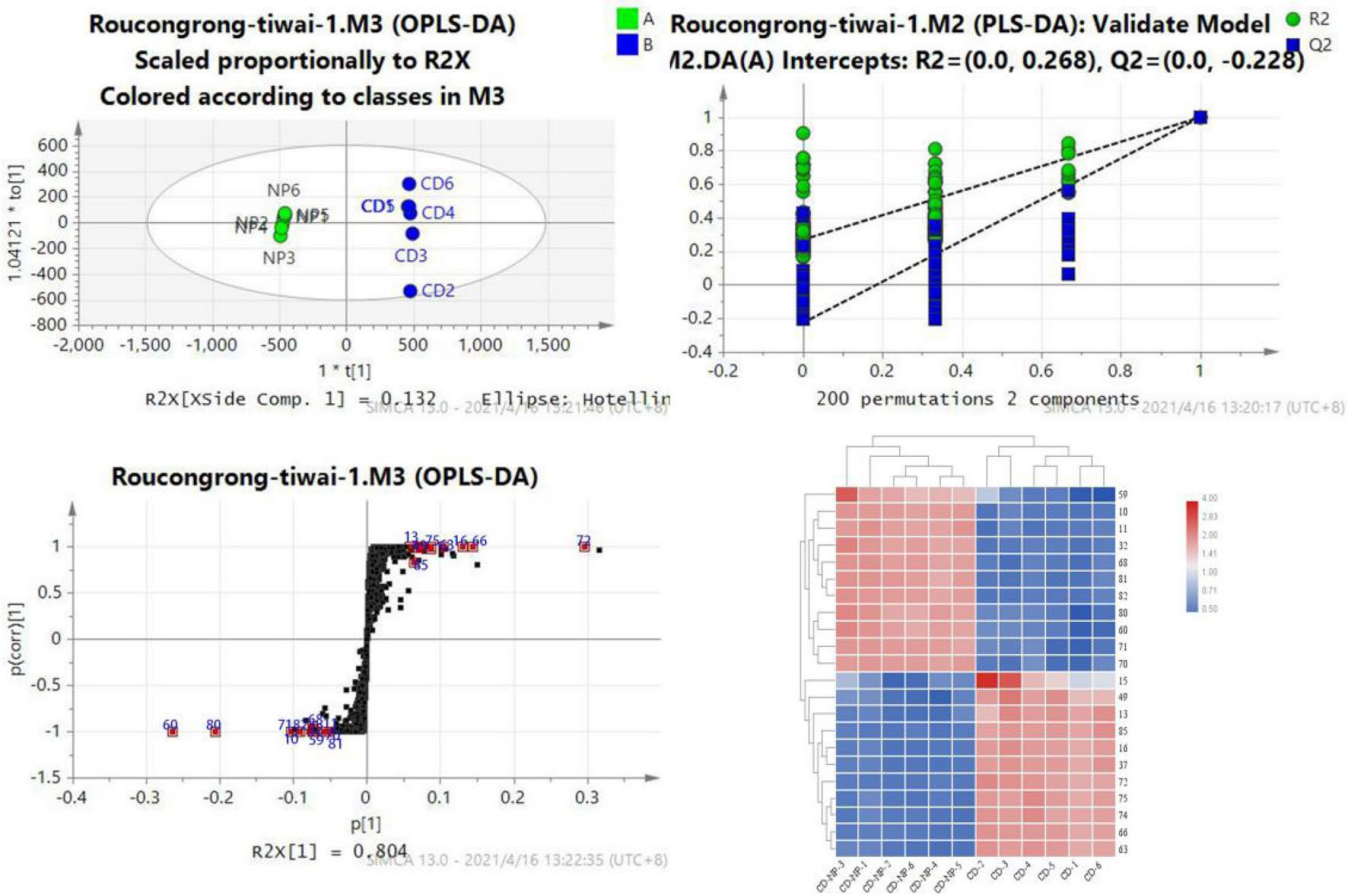
The OPLS-DA /permutation test/ S-plot/heatmaps indicating the intensities of effective biomarkers between CD and CD-NP Compounds 13, 15, 16, 37, 49, 63, 66, 72, 74, 75, and 85 are the differential components of CD, while compounds 10, 11, 32, 59, 60, 68, 70, 71, 80, 81, and 82 are the differential components of CD-NP*.*

**Fig. 7:**
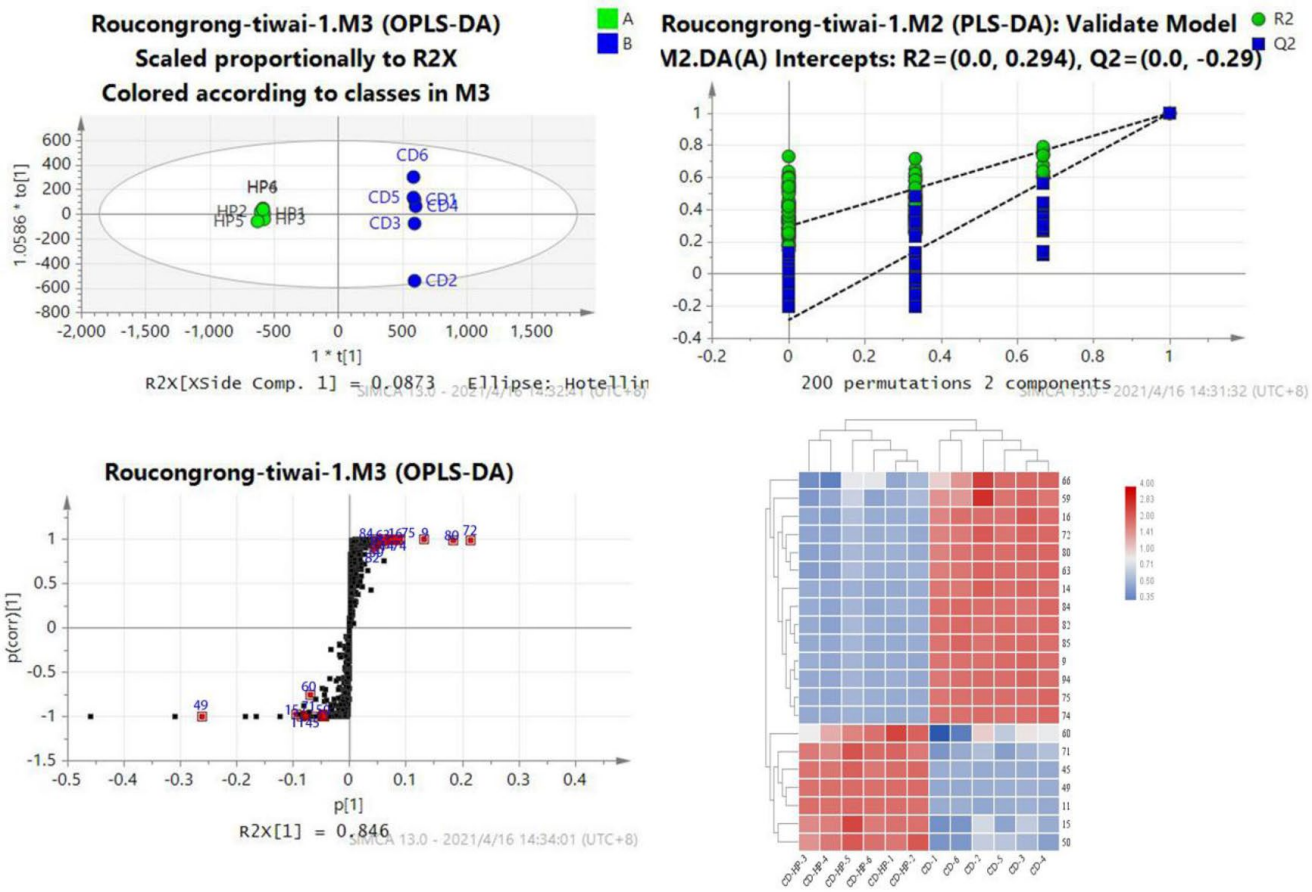
The OPLS-DA/permutation test/S-plot/heatmaps revealing the intensities of effective biomarkers between CD and CD-HP Compounds 9, 14, 16, 59, 63, 66, 72, 74, 75, 80, 82, 84, 85, and 94 are the differential components of CD, and 11, 15, 45, 49, 50, 60, and 71 are the differential components of CD-HP*.*

To distinguish CD from CD-HP and CD-NP, OPLS-DA, permutation test, S-plot, and VIP value were developed. (Figs. 5, 6, 7) The obtained results revealed that many components were key characteristic components of each product. The screening condition was the VIP > 1 and *P* < 0.05. From the date of the S-plot, the characteristic components were evaluated, which were commonly existing in the three groups.

**Fig. 8:**
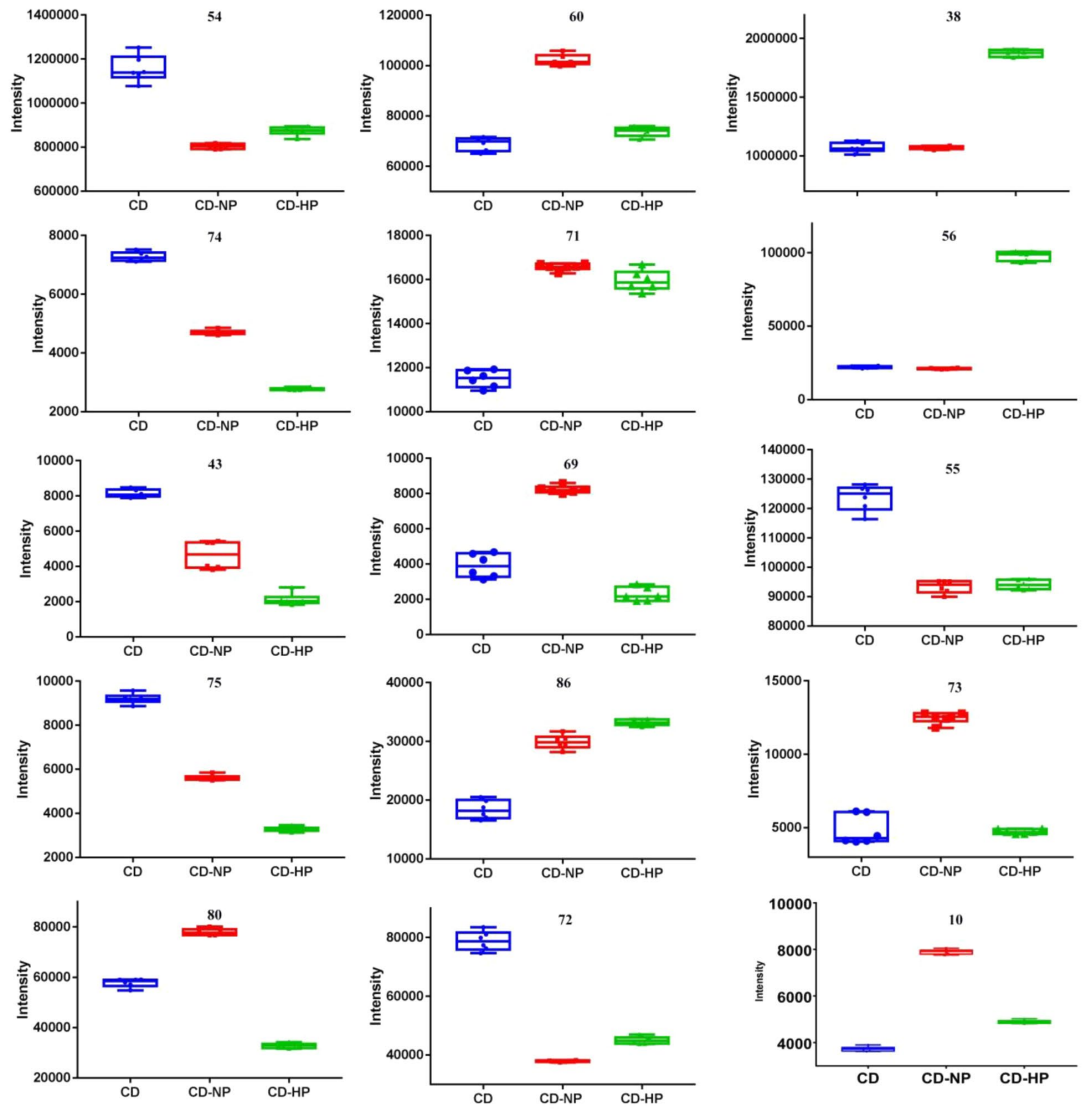
The Intensity of mainly PhGs in CD and its processed products

**Fig. 9:**
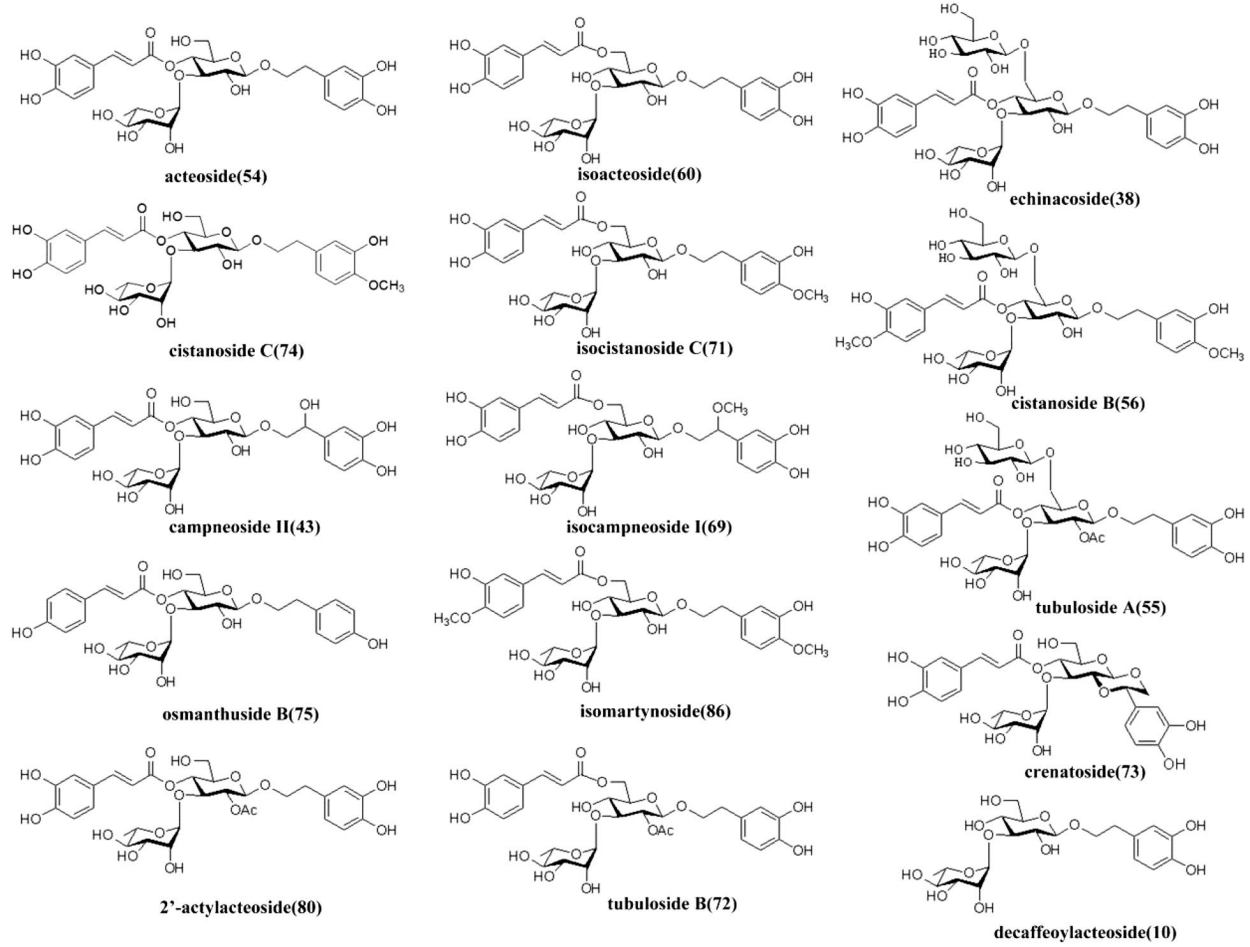
Chemical Structures of mainly PhGs in CD and its processed products.

From Fig. 8, we found the intensity of acteoside (54), cistanoside C (74), campneoside II (43), osmanthuside (75), and 2'-actylacteoside (80) having the 4'-*O*-caffeoyl group in the 8-*O*-β-d-glucopyranosyl part (see Fig. 9) decreased after being processed by rice-wine, while the intensity of isoacetoside (60), isocistanoside (71), isocampneoside I (69), isomartynoside (86) having the 6'-*O*-caffeoyl group (see Fig. 9) increased, especially for the CD-NP group. Though tubuloside B (72) having 6'-*O*-caffeoyl group, the same as isoacteoside, the intensity decreased because of its 2'-actyl group. The intensity of echinacoside (38) and cistanoside B having 6'-*O*-β-d-glucopyranosyl moiety groups increased, but the intensity of tubuloside A (55) decreased also because of its 2'-actyl group.

**Fig. 10:**
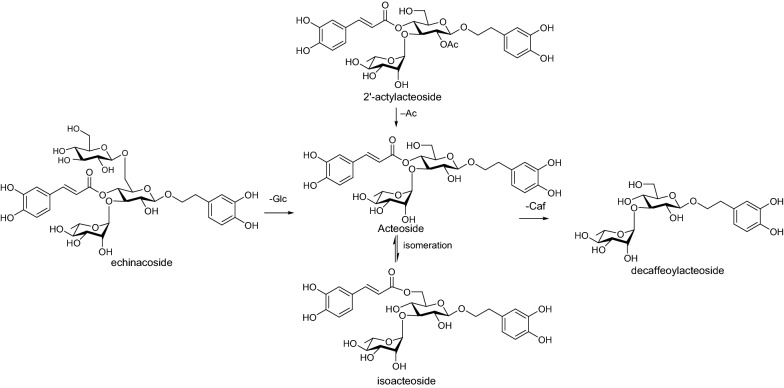
The possible reaction for PhGs during the processing

Our research team also studied the thermal stability of acteoside and isoacteoside, and found that acteoside was unstable in water, methanol and yellow rice wine solution, and could be converted into isoacteoside partly under heating condition. But the thermostability of isoacteoside was better, especially in yellow rice wine solution. Figure 10 showed the possible changes of PhGs in CD during processing:

### Identification of the metabolites in rats

From high-resolution mass spectrometry data, the accurate molecular weight and elemental composition for metabolites and protomolecule compounds were analyzed and compared. As the same kinds of compounds in TCM showed similarity in metabolic modifications, the correlations of phytochemical constituents in vitro can extend to their metabolites in vivo. Meanwhile, based on conventional biotransformation pathways, a reasonable change of molecular weight was inferred. Finally, the metabolites were identified by analyzing the MS^E^ mass spectra of the metabolites and proto-compounds fragmentation pathway in the mass spectrum [21, 22]. Compared with the blank sample, its components were identified in vivo based on the information provided by chromatogram-mass spectrum, the possibility of a metabolic reaction, the characteristics of the compound structure, and the fragmentation rule of its mass spectrum. See Table 3.

**Table 3:** Identified Metabolites in plasma, urine and feces of aqueous extract in CD and its processed products

No.	rtmed	Measured mass	Error (mDa)	Formula	Distribution	Identification	Status
1	0.77	179.0389	4.4	C_9_H_8_O_4_	U(CD, CD-NP, CD-HP) F(CD-NP, CD-HP)	Caffeic acid	Metabolites
2	0.81	149.0653	5.0	C_9_H_9_O_2_	U(CD, CD-NP, CD-HP)	3-phenylpropionic acid	Metabolites
3	0.93	195.0623	-3.5	C_10_H_12_O_4_	U(CD) S(CD-NP) F(CD-HP)	Methylated 3,4-dihydroxybenzenepropionic acid	Metabolites
4	1.02	193.0524	2.3	C_10_H_10_O_4_	U(CD, CD-NP, CD-HP)	Methylated caffeic acid	Metabolites
5	1.18	167.0762	5.4	C_9_H_12_O_3_	U(CD-NP)	Methylated HT	Metabolites
6	3.31	185.117	− 0.8	C_10_H_18_O_3_	U(CD, CD-HP) F(CD-HP)	Ajugol deglycosylation product	Metabolites
7	3.52	167.0536	− 0.9	C_8_H_8_O_4_	U(CD-NP)	HT oxidation	Metabolites
8	4.48	361.1491	− 0.8	C_16_H_26_O_9_	U(CD, CD-NP, CD-HP) F(CD-HP)	Hydroxylated kankanoside A or isomer	Metabolites
9	4.70	541.1144	− 8.3	C_20_H_30_O_15_S	S(CD-NP, CD-HP) F(CD-HP)	Decaffeoylacteoside sulfate conjugation	Metabolites
10	4.73	153.0504	− 4.8	C_8_H_10_O_3_	U(CD, CD-NP, CD-HP) F(CD-NP, CD-HP)	HT	Metabolites
11	4.85	123.0821	1.1	C_8_H_12_O	U(CD, CD-NP, CD-HP) F(CD, CD-HP)	Geniposide hydrolysated product	Metabolites
12	5.14	246.9911	− 1.4	C_8_H_8_O_7_S	U(CD, CD-NP, CD-HP) F(CD-NP)	3,4-dihydroxyphenylacetic acid sulfate conjugation	Metabolites
13	5.23	361.1471	− 2.8	C_16_H_26_O_9_	U(CD, CD-NP, CD-HP) F(CD-NP, CD-HP)	Hydroxylated kankanoside A or isomer	Metabolites
14	5.35	313.0962	3.9	C_14_H_18_O_8_	U(CD, CD-NP, CD-HP) F(CD-NP, CD-HP)	Tyrosol glucuronide conjugation	Metabolites
15	5.63	217.0138	− 3.3	C_8_H_10_O_5_S	U(CD, CD-NP, CD-HP) F(CD-NP, CD-HP)	Tyrosol sufate conjugation	Metabolites
16	5.73	329.0851	− 2.2	C_14_H_18_O_9_	U(CD, CD-NP, CD-HP)	HT-glucuronide conjugation	Metabolites
17	5.98	233.0170	− 5.0	C_8_H_10_O_6_S	U(CD, CD-NP, CD-HP) F(CD, CD-NP, CD-HP)	HT sulfate conjugation	Metabolites
18	6.54	185.1114	− 6.4	C_10_H_18_O_3_	U(CD, CD-NP, CD-HP) F(CD-HP)	Deglycosylated kankanoside N	Metabolites
19	6.76	261.0084	1.5	C_9_H_10_O_7_S	U(CD, CD-NP, CD-HP) F(CD-NP)	3,4-dihydroxybenzenepropionic acid sulfate conjugation	Metabolites
20	7.01	183.1085	6.4	C_10_H_16_O_3_	U(CD, CD-NP, CD-HP)	Deglycosylated kankanoside A or isomer	Metabolites
21	7.16	461.1605	− 5.4	C_20_H_30_O_12_	F(CD-NP)	Decaffeoylacteoside	Proto
22	7.19	247.0278	0.1	C_9_H_12_O_6_S	U(CD, CD-NP, CD-HP)	Methylated HT sulfate conjugation	Metabolites
23	7.28	345.1476	− 7.3	C_16_H_25_O_8_	U(CD, CD-NP, CD-HP) S(CD, CD-NP)	Kankanoside A or isomer	Proto
24	7.57	215.0024	0.2	C_8_H_8_O_5_S	U(CD-HP)	HT sulfate conjugation dehydration product	Metabolites
25	7.69	355.0704	3.9	C_15_H_16_O_10_	U(CD-HP) S(CD)	CA glucuronide conjugation	Metabolites
26	7.78	343.1037	0.8	C_15_H_20_O_9_	U(CD, CD-NP, CD-HP)	Methylated HT glucuronide conjugation	Metabolites
27	7.81	258.994	1.5	C_9_H_8_O_7_S	U(CD, CD-NP, CD-HP)	CA sulfate conjugation	Metabolites
28	8.19	375.1284	− 0.7	C_16_H_24_O_10_	U(CD, CD-NP, CD-HP)	8-epilogani acid	Proto
29	8.52	245.0125	0.5	C_9_H_10_O_6_S	U(CD, CD-NP, CD-HP) F(CD-HP)	3-HPP sulfate conjugation	Metabolites
30	8.53	193.0531	0.8	C_10_H_10_O_4_	U(CD, CD-NP, CD-HP)	Geniposidic acid deglycosylation dehydration product	Metabolites
31	8.90	341.0942	6.9	C_15_H_17_O_9_	U(CD, CD-NP, CD-HP)	3-HPP glucuronide conjugation	Metabolites
32	9.02	242.9951	− 2.1	C_9_H_7_O_6_S	U(CD, CD-NP, CD-HP)	Dehydroxylated CA sulfate conjugation	Metabolites
33	9.06	181.0491	− 1.0	C_9_H_10_O_4_	U(CD, CD-NP, CD-HP) F(CD, CD-NP, CD-HP)	3,4-dihydroxybenzenepropionic acid	Metabolites
34	9.08	151.0352	− 4.3	C_8_H_8_O_3_	U(CD, CD-NP, CD-HP)	Catalpol deglycosylated dehydration product	Metabolites
35	9.58	273.0064	− 0.5	C_10_H_9_O_7_S	U(CD, CD-NP, CD-HP)	Methylated CA sulfate conjugation	Metabolites
36	10.02	275.0209	− 1.6	C_10_H_12_O_7_S	U(CD-NP, CD-HP)	Methoxylated 3-HPP sulfate conjugation	Metabolites
37	10.13	583.1320	− 1.3	C_22_H_32_O_16_S	U(CD, CD-NP, CD-HP)	Cistanoside H sulfate conjugation	Metabolites
38	10.28	299.1108	− 2.3	C_14_H_19_O_7_	U(CD-HP)	Salidroside	Proto
39	10.4	163.04	0.5	C_9_H_8_O_3_	U(CD, CD-NP, CD-HP) S(CD, CD-HP) F(CD, CD-NP, CD-HP)	Dehydroxylated CA	Metabolites
40	10.91	199.0641	3.5	C_9_H_10_O_5_	U(CD-NP)	Catalpol hydrolysated product	Metabolites
41	11.17	521.1816	− 5.4	C_22_H_33_O_14_	U(CD-HP)	6-deoxycatalpol glucuronide conjugation	Metabolites
42	11.29	165.0558	0.6	C_9_H_10_O_3_	U(CD, CD-NP, CD-HP) F(CD, CD-NP, CD-HP)	3-HPP	Metabolites
43	11.31	332.1479	0.8	C_15_H_24_O_8_	U(CD, CD-NP, CD-HP)	Gluroside	Proto
44	11.31	211.0665	5.8	C_10_H_12_O_5_	U(CD, CD-NP, CD-HP) F(CD, CD-NP, CD-HP)	Deglycosylated geniposidic acid	Metabolites
45	12.15	169.0487	− 1.4	C_8_H_8_O_4_	U(CD, CD-NP, CD-HP)	Catalpol deglycosylated product	Metabolites
46	12.15	785.2552	4.8	C_35_H_45_O_20_	F(CD, CD-NP, CD-HP)	Echinacoside	Proto
47	13.66	345.1571	2.2	C_16_H_25_O_8_	U(CD, CD-NP, CD-HP) S(CD-NP)	6-deoxycatapol	Proto
48	13.95	489.1514	− 9.4	C_21_H_29_O_13_	U(CD-HP)	Cistanoside F reduction	Metabolites
49	14.40	487.1480	2.8	C_21_H_27_O_13_	F(CD, CD-NP)	Cistanoside F	Proto
50	14.53	347.1747	− 4.1	C_16_H_27_O_8_	U(CD, CD-HP)	Kankanoside N	Proto
51	14.55	477.1193	− 0.4	C_23_H_26_O_11_	U(CD-HP)	Calceolarisolide A	Proto
52	14.84	315.1174	9.4	C_14_H_20_O_8_	F(CD-NP)	3,4-dihydroxyphenethyl glycoside	Metabolites
53	15.03	197.0833	1.9	C_10_H_13_O_4_	U(CD, CD-NP, CD-HP)	Deglycosylation products of 8-epideoxyloganic acid	Metabolites
54	16.43	230.9984	1.0	C_8_H_8_O_6_S	U(CD, CD-HP) F(CD-NP)	4-phenylacetic acid sulfate conjugate	Metabolites

### Identification of phenylethanol glycosides related metabolites

**Fig. 11:**
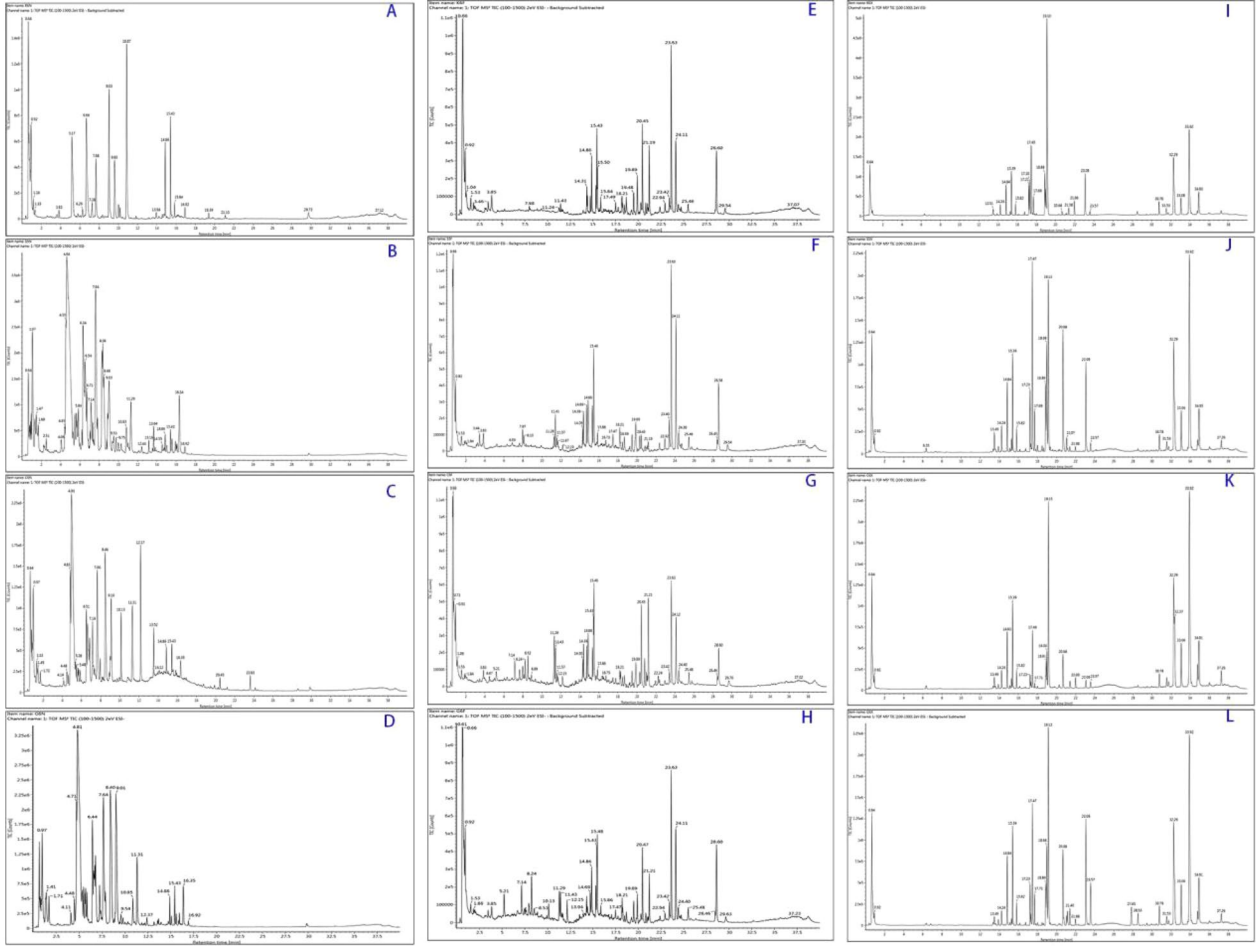
Chromatograph of TIC. **A** Urine sample in BC group; **B** Urine sample in CD group; **C** Urine sample in CD-NP group; **D** Urine sample in CD-HP group; **E** Feces sample in BC group; **F** Feces sample in CD group; **G** Feces sample in CD-NP group; **H** Feces sample in CD-HP group; **I** Plasma sample in BC group; **J** Plasma sample in CD group; **K** Plasma sample in CD-NP group; **M** Plasma sample in CD-HP group

UNIFI platform was used for processing. Figure 11 showed the TIC chromatograph of urine, feces and plasma for CD and its processed products. Compared with blank samples, a total of 54 metabolites were identified in rats, including 10 prototype components and 44 metabolites, in which 24, 49, and 6 were in feces, urine, and plasma, accordingly.

**Fig. 12:**
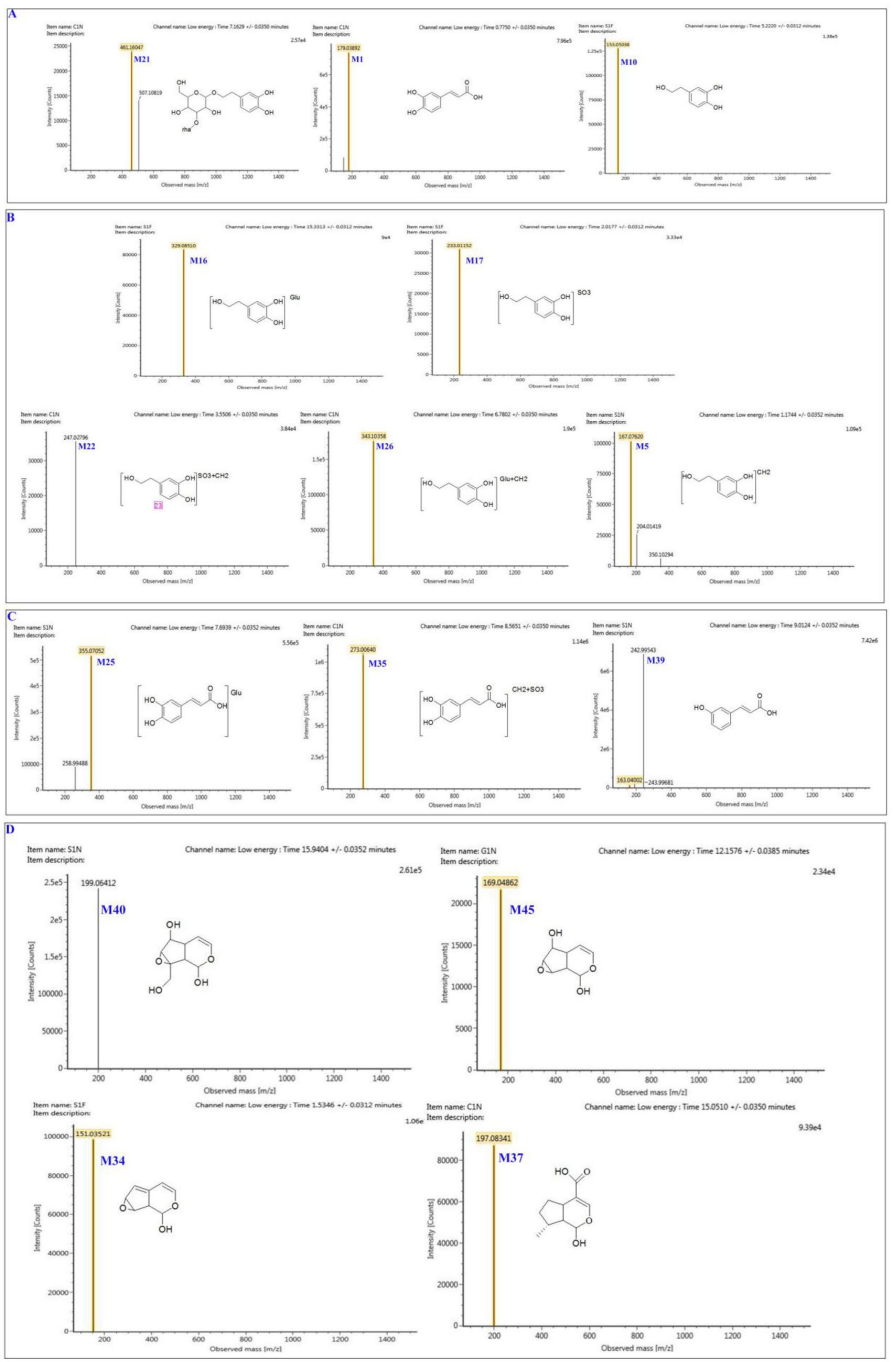
Mass spectrum of some metabolites in CDs

Based on accurate mass, fragmentation cascade, and predictable neutral losses by biotransformation, a total of 35 phenylethanoid glycosides-associated metabolites were tentatively evaluated. The related metabolites of phenylethanoid glycosides have similar mass spectrum fragmentation patterns, like the typical decaffeoyl fragment *m/z* 461.1605, then further hydrolyzed by glycosidic and ester bonds in vivo, and metabolized into hydroxytyrosol (HT) (*m/z* 153.0504, C_8_H_10_O_3_, 4.73 min) and caffeic acid (CA)(*m/z* 179.0389, C_9_H_7_O_4_, 0.77 min), see Fig. 12A.

M11 indicated [M–H]^−^ at *m/z* 153.0504 with formula *i.e.,* C_8_H_10_O_3_, and identified as HT. M16 presented [M–H]^−^ at *m/z* 329.0851, which was 176 Da elevated than that of HT, revealing that it might be a glucuronidated metabolite of HT. The [M–H]^−^ of M26 was at *m/z* 343.1037, 14 Da higher than that of HT-glucuronide. Therefore, M26 was identified as HT-methylated glucuronide. M17 was identified as HT-sulfate based on its [M–H]^−^ at *m/z* 233.0112, 80 Da over the HT, which could be further methylated, then produced M22, which showed the *m/z* 247.0278, indicating that it was HT-methylated sulfated metabolite. M7 (*m/z* 167.0335) and M5 (*m/z* 167.0762) were considered as oxidation products and methylated HT, respectively (Fig. 12B).

M1 indicated [M–H]^−^ at *m/z* 179.0389, elucidated molecular formula was C_9_H_7_O_4_ and identified as caffeic acid (CA). M25 revealed [M–H]^−^ at *m/z* 355.0704, which were 176 Da elevated than that of CA, shows that it might be a glucuronidated metabolite of CA. M27 had *m/z* 258.994, which was 80 Da higher than that of CA, so we elucidated it as CA sulfate, and it could produce M35 (*m/z* 273.0064). As M4 gives the [M–H]^−^ at *m/z* 193.0524, 14 Da higher than CA, it was identified as CA methylated metabolite. M39 was CA dehydroxylation metabolite, with *m/z* 163.04, and it could be sulfated into M32 (*m/z* 242.9951).

M33 (*m/z* 181.0491, C_9_H_10_O_4_, 9.06 min) was the reduction product of CA, that is 3,4-dihydroxybenzenepropionic acid, which could be methylated into M19 (*m/z* 195.0623, C_10_H_12_O_4_, 0.93 min). M33 could be dehydroxyed into M43, that is 3-HPP (*m/z* 165.0558, C_9_H_10_O_3_, 11.29 min), and M31 (*m/z* 341.0942, C_15_H_17_O_9_, 8.90 min) and M29 (*m/z* 245.0125, C_9_H_10_O_6_S, 8.52 min) were the glucuronidated and sulfated products (Fig. 12C).

**Fig. 13:**
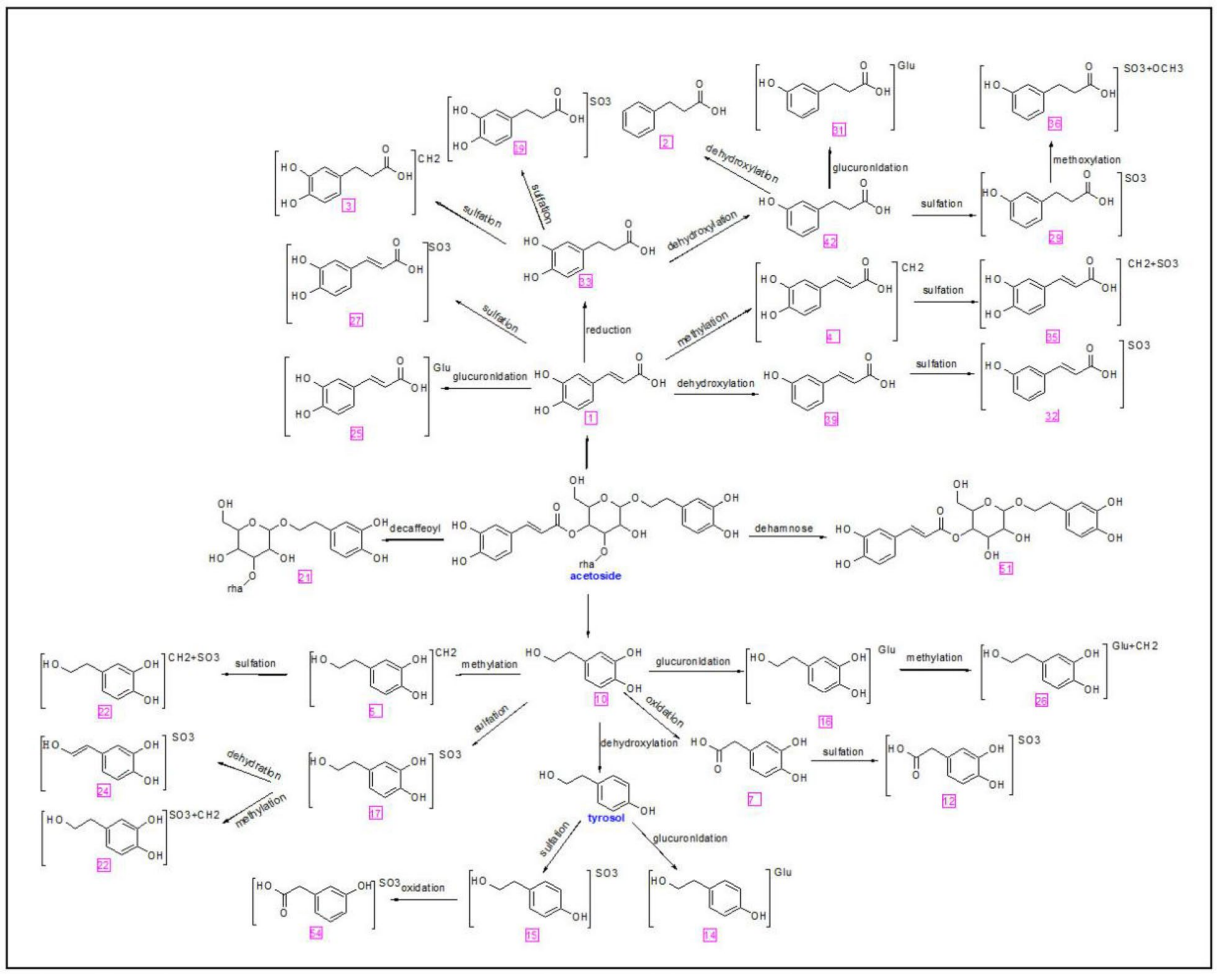
Possible Metabolic pathway of phenylethanoids

For the phenylethanoid glycosides-associated metabolites, the key metabolic cascades were phase II metabolic reactions, *i.e.,* glucuronidation, methylation, and sulfation. The proposed metabolic cascades of phenylethanoids are depicted in Fig. 13.

### Identification of iridoids related metabolites

By analyzing the elemental composition of the metabolites, MS^E^ fragmentation, and associated literature, a total of 19 iridoid-associated metabolites were tentatively evaluated. Iridoid glycosides were hydrolyzed by glycosidic bonds to form their corresponding aglycones. The *m/z* 185.117 was for M8, 162 Da less than ajugol, which was yielded by the loss of glucose residue.

M40 (*m/z *199.0641, Rt 10.91 min) was the deglycosylated product of catalpol. M45 *m/z* 169.0487, Rt 12.15 min) was less than 30 Da that of catalpol deglycosylated metabolite, and was identified as removal of a molecule of CH_2_O metabolite. M34 (*m/z* 151.0352, Rt 9.08 min), was further loss of H_2_O metabolite.

M44 (*m/z* 211.0665, Rt 11.31 min) was a deglycosylated metabolite of geniposide, and M37 (*m/z* 197.0833, Rt 15.03 min) was deglycosylation of 8-epideoxyloganic acid. Metabolic reactions for iridoids could be revealed as phase I metabolism of deglycosylation (Fig. 12D).

### Comparison of metabolic profiling in plasma, urine, and feces between CD and its processed products

2 prototypes in plasma, 7 in urine, and 3 in feces were compared. There were 7 prototypes absorbed in CD, 7 prototypes absorbed in CD-NP, and 8 prototypes in CD-HP. M21 was only detected in the feces group of CD-NP, and M38 and M51 were detected just in urine groups of CD-HP. Compared with metabolites, identical metabolites in plasma, urine, and feces were 4, 42, and 21, respectively. There were 34 metabolites absorbed in the CD group, 39 in CD-NP, and 40 in the CD-HP group. M5, M7, M40, and M52 were only detected in CD-NP groups, while M24, M41, and M48 were just detected in CD-HP groups.

**Fig. 14:**
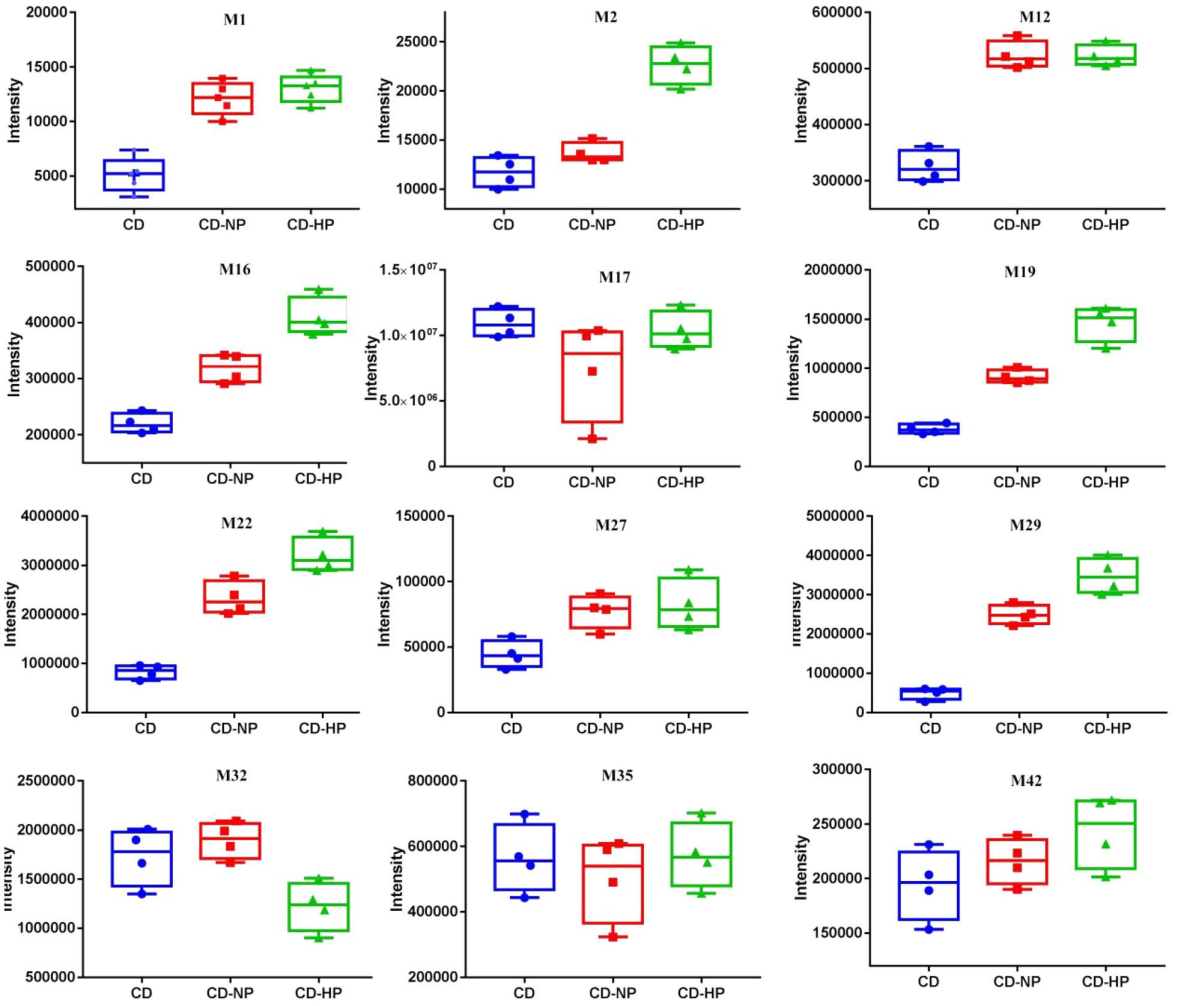
Intensity of main metabolites in urine

Variations were observed in the absorption as well as the metabolism of active compounds in diverse processed products of CD. From Fig. 14, we found that the intensity of HT-sulfate conjugation (M17) was the highest in the urine, followed by 3-HPP sulfate conjugation (M29), methylated HT sulfate conjugation (M22), dehydroxylated CA sulfate conjugation (M32), and 3,4-dihydroxy benzenepropionic acid sulfate conjugation (M19). The content of metabolic products in the processed group was higher than in the CD group, especially for M22, M29, M27, M16, M19, M1, M2. Their precursor compounds, such as hydroxytyrosol have anti-tumor, anti-inflammatory, antibacterial, an tiviral, and antifungal properties [23]. Caffeic acid possesses anti-inflammatory, anti-cancer, and antiviral activities [24]. It was consistent with the clinical use of CD and its processed products.

## Discussion

CD is a TCM, and its major bioactive components, including PhGs, iridoids, polysaccharide have been documented by various research studies. In TCM clinical practice, the processed products of CD have been widely used relative to raw ones. The chemical composition will be changed during the processing, which may lead to changes in the medicinal effects (Fig. 14).

PhGs are a type of phenolic compound characterized by a β-glucopyranoside structure bearing a hydroxyphenylethyl moiety as the aglycone. These compounds often comprise caffeic acid and rhamnose attached to the glucose residue through ester or glycosidic linkages respectively. In the current study, the qualitative analyses of CD, CD-NP, and CD-HP were carried out, and a total of 97 compounds, including phenylethanoid glycosides (PhGs), iridoids, etc. were identified. The obtained results showed the variations in chemical composition before and after processing. The intensity of PhGs having the 4'-*O*-caffeoyl group in the 8-*O*-β-d-glucopyranosyl part, like acteoside, cistanoside C, campneoside II, osmanthuside decreased after being processed, while PhGs with 6'-O-caffeoyl group in the 8-*O*-β-d-glucopyranosyl part, such as isoacetoside, isocistanoside, isocampneoside I, isomartynoside increased, especially in the CD-NP group. The intensity of echinacoside and cistanoside B whose structure possess 6'-*O*-β-d-glucopyranosyl moiety also increased. PhGs having 2'-actyl group often decreased because of hydrosis reaction during the process, like tubuloside B, 2-acetylacteoside.

Investigation of metabolites absorbed in vivo was carried out after oral administration of CD and its processed products. The metabolic processes of phase II were the key cascades and most of the metabolites were sulfate, glucuronide, and methylated conjugates. Phenylethanol glycosides have low oral absorption and utilization. They are difficult to be absorbed into the blood, and act as progenitors to play their roles after metabolic activation in vivo. Phenylethanoids produced into phenylethanolaglycone, like hydroxytyrosine (HT) and caffeic acid (CA) and its derivative 3-hydroxyphenylpropionic acid (3-HPP), these metabolites may be more easily absorbed into the plasma and have a better medicinal effect.

Most of the metabolites were found in their lower concentrations or not detected in rat plasma, however, higher concentration was observed in the urine, indicating that metabolites would get easily eliminated via urine. As depicted in Table 3, the same compounds were determined in various groups, while considerable variations were found in the concentrations of the metabolites which might be associated with the unequal efficacy of CD and its processed products. HT-sulfate conjugation (M17) have the highest intensity in the urine, followed by 3-HPP sulfate conjugation (M29), methylated HT sulfate conjugation (M22), dehydroxylated CA sulfate conjugation (M32), and 3,4-dihydroxy benzenepropionic acid sulfate conjugation (M19). The content of metabolic products in the processed group was higher than in the CD group, especially for M22, M29, M27, M16, M19, M1, M2.

Generally, the components having high exposure in target organs could be effective. A sufficient amount of phenylethanoids and their derivatives have been evaluated and determined in vitro. Acteoside is the characteristic compounds, whose content decreased after being processed by rice-wine, and the content of isoacteoside, isocistanoside C, isocampneoside I increased correspondingly. The degradation products of PhGs, like CA and HT derivatives could be evaluated in the bio-samples, and rice-wine processing can enhance the absorption of metabolites in vivo.

## Conclusion

In this study, 97 compounds were detected in the extracts of CD and its processed product. The degradation of few glycosides occurred under an elevated temperature and as a result, some new isomers and complexes were synthesized. In in vivo study, prototype components (10) and metabolites (44) were determined or tentatively evaluated in rat plasma, feces, and urine. Phase II metabolic processes were the key cascades, most of the metabolites were associated with echinacoside or acteoside, like HT, CA and their derivatives 3-hydroxyphenylpropionic acid 3-HPP. These metabolites may be more easily absorbed into the plasma and have a better medicinal effect. The obtained results showed that the chemical composition of CD was different and affected the disposition of the compound in vitro and in vivo.

## Data Availability

The datasets used and/or analyzed during the current study are available from the corresponding author on reasonable request.

## Abbreviations

PhGs: Phenylethanoid glycosides
CD: *Cistanche deserticola*
CMM: Chinese Materia Medica
TCM: Traditional Chinese Medicine
CD-NP: *Cistanche deserticola* Processed by steaming with rice-wine under normal pressure
CD-HP: *Cistanche deserticola* Processed by steaming with rice-wine under high pressure
UPLC-Q-TOF-MS^E^: Ultra-high performance liquid chromatography coupled with TOF-MS^E^
PCA: Principal component analysis
VIP: Variable importance for the projection
CA: Caffeic acid
HA: Hydroxytyrosol
